# EP300‐Mediated MTF1‐K218 Lactylation Buffers AR‐Driven Copper Overload to Suppress Cuproptosis in Castration‐Resistant Prostate Cancer

**DOI:** 10.1002/advs.77110

**Published:** 2026-08-11

**Authors:** Kai Li, Yong Wei, Yongshan Li, Yuxiang Dong, Jiancheng Lv, Mingzhi Yuan, Ao Shen, Fuyang Liu, Yetao Zhang, Youjian Li, Ruixi Yu, Yang Liu, Tong Zhao, Jun Wang, Kai Zhou, Zongyao Fan, Manning Wang, Jun Xu, Mulong Du, Min Gu, Bing Yao, Qingyi Zhu

**Affiliations:** ^1^ Department of Urology Jiangsu Key Laboratory of Urological Disease Prevention and Treatment The Second Affiliated Hospital of Nanjing Medical University Nanjing Medical University Nanjing China; ^2^ Department of Urology First Affiliated Hospital School of Medicine Zhejiang University Hangzhou China; ^3^ Jiangsu Key Laboratory of Intelligent Medical Image Computing School of Artificial Intelligence Nanjing University of Information Science and Technology Nanjing China; ^4^ Digital Medical Research Center School of Basic Medical Sciences Fudan University Shanghai China; ^5^ Shanghai Key Laboratory of Medical Image Computing and Computer Assisted Intervention Fudan University Shanghai China; ^6^ Department of Urology Jiangnan University Medical Center Wuxi China; ^7^ Department of Pathology Wuxi People's Hospital Wuxi China; ^8^ Department of Urology Jinling Hospital Affiliated Hospital of Medical School Nanjing University Nanjing China; ^9^ Department of Biostatistics Center for Global Health School of Public Health Nanjing Medical University Nanjing China; ^10^ National Experimental Teaching Center of Basic Medical Science Department of Medical Genetics School of Basic Medical Sciences Nanjing Medical University Nanjing China; ^11^ State Key Laboratory Cultivation Base of Biomarkers for Cancer Precision Prevention and Treatment Collaborative Innovation Center for Cancer Personalized Medicine NHC Key Laboratory of Antibody Technique Jiangsu Province Engineering Research Center of Antibody Drug Nanjing Medical University Nanjing China

**Keywords:** androgen receptor, cancer research, copper toxicity, copper, cytosol, downregulation and upregulation, metallothionein, mitochondrion, prostate cancer, transcription factor

## Abstract

Inducing cuproptosis for cancer therapy currently relies on supraphysiological copper or copper ionophores. Although serum copper is elevated in patients with prostate cancer, whether this is sufficient to trigger physiological cuproptosis remains unclear. Here, we show that tumor copper levels positively correlate with androgen receptor (AR) activity, and castration‐resistant prostate cancer (CRPC) with hyperactivated AR exhibits pathological copper accumulation. AR activation enhances copper uptake while simultaneously conferring tolerance to copper toxicity, creating a buffered copper state. This adaptive response is mediated by metal‐responsive transcription factor 1 (MTF1), which is transcriptionally upregulated by AR and undergoes EP300‐dependent lactylation of lysine 218, promoting copper‐induced nuclear translocation. Nuclear MTF1 activates metallothioneins (MT1E, MT1F, and MT1M) that sequester cytosolic copper and restrict mitochondrial copper accumulation. Disrupting MTF1 collapses this buffering system, enabling endogenous copper to trigger cuproptosis and suppress CRPC growth. These findings identify cuproptosis as a therapeutically exploitable vulnerability in CRPC.

## Graphical abstract

Androgen receptor (AR) drives copper accumulation in prostate cancer while inducing MTF1 to buffer copper toxicity. EP300‐mediated lactylation of MTF1 at K218 promotes its nuclear translocation and metallothionein expression, sequestering cytosolic copper and preventing mitochondrial cuproptosis. Disrupting this MTF1‐dependent buffering system unleashes cuproptosis and suppresses tumor growth in castration‐resistant prostate cancer.

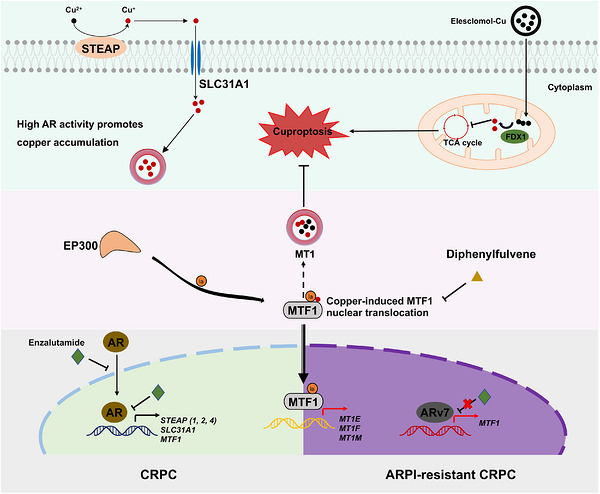

## 1 Introduction

Prostate cancer (PCa) is one of the most prevalent malignancies in men worldwide and is projected to rank first in incidence and second in mortality among male cancers in the United States in 2026 [1]. Since the seminal work by Huggins and Hodges established the androgen dependence of PCa [2], androgen deprivation therapy (ADT) has remained a central treatment strategy [3]. Despite initial responses, most patients progress to castration‐resistant prostate cancer (CRPC) within 2–3 years [4], a state frequently driven by persistent androgen receptor (AR) signaling [5, 6, 7]. Androgen receptor pathway inhibitors (ARPI), including enzalutamide (ENZ), have been widely used to treat CRPC by blocking AR activation and transcriptional output [8, 9]. However, resistance is nearly universal [10, 11], highlighting the need for therapeutic strategies that target vulnerabilities downstream of hyperactivated AR signaling.

Copper (Cu) is an essential trace element that supports cell growth and metabolism by serving as a cofactor for key enzymes, yet excessive copper is cytotoxic [12, 13, 14, 15, 16]. The discovery of cuproptosis—a copper‐dependent form of regulated cell death triggered by mitochondrial copper accumulation—has renewed interest in targeting copper metabolism for cancer therapy [17, 18]. Within mitochondria, Cu^2+^ is reduced to Cu^+^ by ferredoxin 1 (FDX1), leading to destabilization of iron–sulfur cluster (Fe–S) proteins and aggregation of lipoylated proteins, which ultimately induces cell death. Cuproptosis has attracted growing attention across diverse malignancies [18, 19]. However, current studies have primarily relied on artificial systems in which cells are exposed to copper ionophores or supraphysiological copper concentrations [20, 21, 22], leaving unresolved whether cuproptosis can occur under endogenous, physiological copper levels within tumors. In prostate cancer, serum copper levels are elevated, and androgen stimulation enhances cellular copper uptake [23, 24], suggesting that tumors with high AR activity may exhibit increased copper demand and accumulation. Whether such copper enrichment predisposes prostate cancer cells to endogenous cuproptosis, and how AR signaling coordinates copper uptake, buffering, and toxicity, remain poorly understood.

Metal regulatory transcription factor 1 (MTF1) is a mediator of cellular metal stress responses, rapidly translocating to the nucleus upon metal exposure to induce metallothionein (MT) expression [25, 26, 27, 28, 29]. The transcriptional activity of MTF1 is regulated by post‐translational modifications (PTMs), including phosphorylation and SUMOylation [30, 31]. In this study, we identify MTF1 as a key regulator that sustains copper homeostasis in CRPC. AR activation promotes copper uptake while transcriptionally upregulating MTF1, thereby enhancing MT1 expression to chelate excess copper. Moreover, the lactate‐enriched tumor microenvironment in CRPC drives EP300‐dependent lactylation of MTF1 at lysine 218, which further facilitates its copper‐induced nuclear translocation and enhances MT1 transcription. Importantly, by exploiting the AR‐driven high‐copper state in CRPC, targeting MTF1 disrupts this buffering system and triggers cuproptosis independent of exogenous copper supplementation. These findings establish MTF1 as a critical node linking AR signaling, copper homeostasis, and cuproptosis resistance, and suggest that targeting MTF1 represents a promising therapeutic strategy for CRPC, including ARPI‐resistant disease.

## 2 Results

### 2.1 AR Drives Copper Accumulation in Prostate Cancer

**FIGURE 1:**
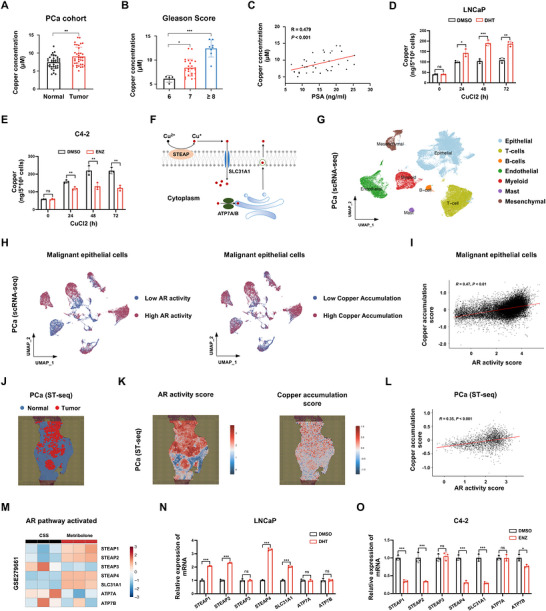
AR drives copper accumulation in prostate cancer. (A) Measurement of copper concentration in prostate tumors and paired adjacent normal tissues (36 pairs). (B) Copper concentration in tumor tissues with different Gleason scores. (C) Correlation analysis between patient prostate‐specific antigen (PSA) concentration and tumor tissue copper concentration using Spearman's analysis. (D) Cellular copper content in LNCaP cells following AR activation with DHT (10 µm) and Cu supplementation (1 µm). (E) Cellular copper content in C4‐2 cells after AR inhibition with ENZ (10 µm). Cells were cultured in medium supplemented with Cu (1 µm). (F) Schematic diagram of copper uptake and export. (G) UMAP plot of cells from ten prostate cancer cases, colored by assigned cell type based on scRNA‐seq. (H) UMAP visualization of scRNA‐seq data from prostate cancer, showing clustering and the distribution of high versus low AR activity (inferred using AR pathway gene signatures) and copper accumulation scores (calculated as copper uptake minus export) within malignant epithelial cells. (I) Spearman's correlation analysis of the association between AR activity score and copper accumulation score in scRNA‐seq data. (J) Distribution map of tumor and normal regions in ST‐seq. (K) Analysis based on ST‐seq of PCa. Copper accumulation score = AddModuleScore (copper uptake: STEAP1–4, SLC31A1) – AddModuleScore (copper export: ATP7A, ATP7B). AR activity score = AddModuleScore (AR pathway: KLK3, KLK2). (L) Association between AR activity score and copper accumulation score analyzed by Spearman's correlation analysis in ST‐seq data. (M) Heatmap showing RNA‐seq expression of STEAP1–4, SLC31A1, and ATP7A/B upon AR activation (Metribolone, 1 nM, 48 h). (N) mRNA expression of STEAP1–4, SLC31A1, and ATP7A/B in AR‐activated LNCaP cells treated with DHT (10 µm). (O) mRNA expression of STEAP1–4, SLC31A1, and ATP7A/B in AR‐inhibited C4‐2 cells treated with ENZ (10 µm). All graphic data are presented as mean ± SD (*n* = 3 replicates) from one representative of three independent experiments unless otherwise specified. Statistical analysis by Student's *t*‐test (A, D, E, N, O). Comparisons among multiple groups were performed by one‐way ANOVA followed by Tukey's multiple comparisons test (B). ns: no significance, ^*^ *p* < 0.05, ^**^ *p* < 0.01, ^***^ *p* < 0.001.

To assess whether AR activity is linked to copper dysregulation in prostate cancer, we examined copper levels in paired tumor and adjacent tissues. Analysis of 36 paired samples revealed significant copper enrichment in tumors, which increased with Gleason grade (Figure 1A,B). Tumor copper levels also showed a positive correlation with serum prostate‐specific antigen (PSA), a clinical surrogate of AR activity (Figure 1C). To determine whether AR directly regulates copper uptake, we treated AR‐positive LNCaP cells with the AR agonist dihydrotestosterone (DHT) and CRPC‐derived C4‐2 cells with the AR antagonist ENZ. AR modulation was confirmed by changes in KLK3 (the gene encoding PSA) expression (Figure S1A–C). DHT significantly increased copper uptake in LNCaP cells, whereas ENZ reduced copper levels in C4‐2 cells, as measured by inductively coupled plasma mass spectrometry (ICP‐MS) (Figure 1D,E; Figure S1D).

Cellular copper levels are controlled by coordinated uptake and export (Figure 1F). To evaluate whether AR activity is linked to copper accumulation at the transcriptional level, we derived a copper‐accumulation score from single‐cell RNA sequencing (scRNA‐seq) data. This score was calculated as the difference between the uptake module (STEAP1–4 and SLC31A1) and the export module (ATP7A and ATP7B) using the AddModuleScore function, and represents a transcriptionally inferred index of net copper retention rather than a direct measurement of copper content. We first performed clustering of all cells (Figure 1G; Figure S1E). Within the epithelial cell populations, we observed a positive correlation between AR activity and the copper‐accumulation score (Figure S1F,G). After further extracting malignant epithelial cells from the epithelial compartment (Figure S1H,I), this positive correlation remained evident in the malignant epithelial populations (Figure 1H, I). To spatially resolve this relationship, we performed spatial transcriptomics on human prostate cancer specimens (Figure 1J; Figure S1J). Notably, AR activity displayed a spatial distribution pattern that was concordant with the copper‐accumulation scores, showing a positive correlation between the two parameters (Figure 1K,L).

Then, we asked whether AR transcriptionally regulates copper transport machinery. RNA‐seq revealed that AR activation selectively upregulated the copper reductases STEAP1, STEAP2, and STEAP4, as well as the copper importer SLC31A1, whereas copper exporters ATP7A and ATP7B were largely unaffected (Figure 1M). These findings were validated by qRT‐PCR and Western blot following DHT or ENZ treatment (Figure 1N,O; Figure S2A,B). ChIP‐qPCR performed in C4‐2 cells validated AR binding to these loci, confirming the specificity of the interaction (Figure S2C–F). Publicly available AR ChIP‐seq data from VCaP cells (GSE252897) further revealed AR binding peaks at the promoter regions of STEAP1, STEAP2, STEAP4, and SLC31A1, and this enrichment was markedly reduced upon AR inhibition (Figure S2G–J). Together, these data demonstrate that AR transcriptionally reprograms copper transport pathways, driving copper accumulation in prostate cancer.

### 2.2 AR Activation Confers Tolerance to Copper‐Induced Cytotoxicity

**FIGURE 2:**
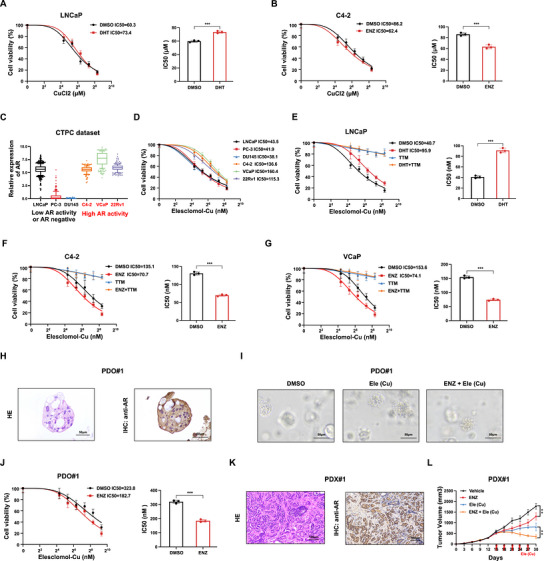
High AR activity mitigates copper toxicity. (A) Relative cell viability of AR‐activated LNCaP cells (DHT, 10 µM) treated with Cu for 48 h. (B) Relative cell viability of AR‐inhibited C4‐2 cells (ENZ, 10 µm) treated with Cu for 48 h. (C) mRNA expression of AR in the six prostate cancer cell lines from the CTPC dataset. (D) Relative cell viability of the six prostate cancer cell lines treated with Ele–Cu for 48 h. (E) Relative cell viability of DHT‐activated LNCaP cells (DHT, 10 µm) treated with Ele–Cu for 48 h, rescued by TTM (1 µm). (F) Relative cell viability of ENZ (10 µm)‐treated C4‐2 cells treated with Ele–Cu for 48 h, rescued by TTM (1 µm). (G) Relative cell viability of ENZ (10 µm)‐treated VCaP cells treated with Ele–Cu for 48 h, rescued by TTM (1 µm). (H) HE staining and anti‐AR immunohistochemistry (IHC) in PDO#1. (I) Bright‐field images of PDO#1 following 48 h treatment with Ele–Cu (300 nm) and ENZ (10 µm). Scale bar, 50 µm. (J) Relative cell viability of AR‐inhibited (ENZ 10 µm) organoids treated with Ele–Cu for 48 h. (K) HE staining and IHC (anti‐AR) of the established PDX tumor tissue. Scale bar, 100 µm. (L) Growth curve of PDX mice treated with Ele–Cu (10 mg/kg, intratumoral injection, every three days starting from day 15) and ENZ (10 mg/kg, oral gavage, daily starting from day 12) (*n* = 5 mice per group). All graphic data are presented as mean ± SD (*n* = 3 replicates) from one representative of three independent experiments unless otherwise specified. Student's *t*‐test was used for A, B, E, F, G, and J. One‐way ANOVA followed by Tukey's multiple comparisons test was used for L. ^**^ *p* < 0.01, ^***^ *p* < 0.001.

In vitro, copper treatment induced marked cytotoxicity in prostate cancer cells, which was rescued by the copper chelator tetrathiomolybdate (TTM) (Figure S3A,B). Paradoxically, despite higher intracellular copper levels, AR‐activated LNCaP cells exhibited increased tolerance to copper‐induced cytotoxicity (Figure 2A). In contrast, AR inhibition not only reduced copper uptake but also exacerbated copper‐induced cell death (Figure 2B). These findings indicate that although AR signaling promotes copper accumulation, it concurrently activates protective mechanisms that buffer copper stress.

To exclude the contribution of differential copper uptake resulting from distinct AR activities, we used the copper ionophore elesclomol (Ele) to deliver equivalent amounts of copper into cells. Under these conditions, CRPC cells with high AR activity (C4‐2, VCaP, and 22Rv1) displayed greater resistance to Ele–Cu–induced cuproptosis (Figure 2C,D). Gain‑of‑function experiments showed that DHT treatment (Figure 2E; Figure S3C) or AR overexpression enhanced resistance to Ele–Cu in LNCaP cells (Figure S3D–G). Conversely, loss‑of‑function approaches robustly sensitized cells to copper‑induced cell death. In C4‑2 cells, both ENZ treatment and AR knockdown significantly increased sensitivity to Ele–Cu (Figure 2F; Figure S3H–L). Similarly, AR inhibition by ENZ in VCaP cells, another AR‑positive cell line with high AR activity, also markedly sensitized cells to cuproptosis (Figure 2G; Figure S3M). In all cases, TTM rescued cell viability, confirming that the observed cytotoxicity was cuproptosis. To evaluate the clinical relevance of these findings, we examined patient‐derived organoid (PDO) and xenograft (PDX). ENZ significantly sensitized PDO#1 derived from a cT2C patient to cuproptosis (Figure 2H–J; Figure S3N,O), and combined ENZ and Ele–Cu treatment markedly suppressed tumor growth in the PDX model (Figure 2K,L; Figure S3P,Q).

Because cuproptosis is initiated in mitochondria, we next examined whether AR modulates mitochondrial responses to copper stress. Binding of copper to mitochondrial lipoylated proteins triggers oligomerization of lipoylated DLAT (Lip‐DLAT), accompanied by a reduction in Lip‐DLAT protein levels. Under Ele–Cu treatment, AR activation markedly attenuated copper‐induced DLAT oligomerization and increased FDX1 and Lip‐DLAT protein levels (Figure S4A), whereas AR knockdown produced the opposite effect (Figure S4B). Notably, AR manipulation did not alter FDX1 or DLAT mRNA expression under copper stress (Figure S4C,D), indicating that these effects are independent of transcriptional regulation. Importantly, in the absence of exogenous copper stress, neither AR activation nor inhibition affected the mRNA or protein levels of FDX1 and Lip‐DLAT (Figure S4E–H), excluding a direct regulatory role of AR on the core cuproptosis machinery. Graded copper exposure alone was sufficient to reduce FDX1 and Lip‐DLAT abundance (Figure S4I,J), in line with previous reports that Cu^+^ destabilizes Fe–S cluster–containing proteins in mitochondria [17]. Collectively, these findings indicate that AR does not directly regulate cuproptosis components, but instead mitigates copper‐induced mitochondrial protein destabilization, most likely by limiting mitochondrial copper accumulation, thereby enabling prostate cancer cells to tolerate elevated intracellular copper and establishing a buffered copper state.

### 2.3 AR Promotes Cuproptosis Resistance by Inducing MT1 Family Proteins

**FIGURE 3:**
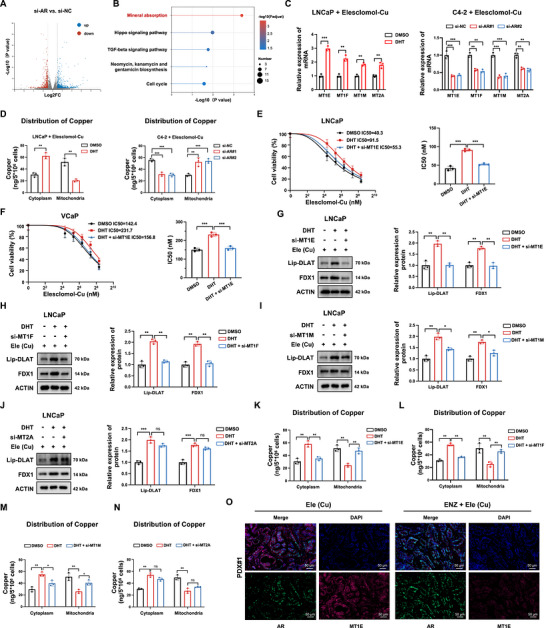
AR promotes MT1 accumulation to suppress cuproptosis. (A and B) Volcano plot (A) and KEGG pathway enrichment analysis (B) from RNA‐seq of AR‐knockdown C4‐2 cells treated with Ele–Cu (100 nm, 12 h). (C) mRNA expression of MT1E, MT1F, MT1M, and MT2A in AR‐activated LNCaP cells (Left) and AR‐knockdown C4‐2 cells (Right), both treated with Ele–Cu (100 nm, 12 h). (D) Mitochondrial and cytoplasmic copper content in LNCaP cells after AR activation (Left) and in C4‐2 cells after AR knockdown (Right), both treated with Ele–Cu (100 nm, 12 h). (E) Relative cell viability of AR‐activated LNCaP cells following knockdown of MT1E and treated with Ele–Cu for 48 h. (F) Relative cell viability of AR‐activated VCaP cells following knockdown of MT1E and treated with Ele–Cu for 48 h. (G–J) Protein levels of Lip‐DLAT and FDX1 in AR‐activated LNCaP cells following knockdown of MT1E (G), MT1F (H), MT1M (I), or MT2A (J) and exposure to Ele–Cu (100 nm, 12 h). (K–N) Mitochondrial and cytoplasmic copper content in LNCaP cells with AR activation followed by knockdown of MT1E (K), MT1F (L), MT1M (M), or MT2A (N), and treated with Ele–Cu (100 nm, 12 h). (O) mIF of AR and MT1E in PDX#1 tumor tissue. Scale bar 50 µm. All graphic data are presented as mean ± SD (n = 3 replicates) from one representative of three independent experiments. Student's *t*‐test was used for (C) left and (D) left. One‐way ANOVA followed by Tukey's multiple comparisons test was used for (C) right, (D) right, and (E–N). ns: no significance, ^*^ *p* < 0.05, ^**^ *p* < 0.01, ^***^ *p* < 0.001.

To elucidate how AR confers resistance to cuproptosis, we performed RNA‐seq in AR‐knockdown C4‐2 cells treated with Ele–Cu (Figure 3A). KEGG pathway analysis revealed prominent enrichment of the mineral absorption pathway (Figure 3B). Among the most significantly downregulated genes, MT1E, MT1F, MT1M, and MT2A encode metallothioneins—cytosolic copper‐binding proteins that could buffer intracellular copper. qRT‐PCR confirmed that DHT stimulation markedly increased, whereas AR knockdown reduced, the expression of these genes (Figure 3C).

To determine whether AR alters intracellular copper distribution, we fractionated cytosolic and mitochondrial compartments and quantified copper by ICP–MS. AR activation promoted copper retention in the cytosol while reducing mitochondrial copper, whereas AR inhibition had the opposite effect (Figure 3D). Functionally, silencing MT1E, MT1F, or MT1M—particularly MT1E—partially restored cuproptosis sensitivity under AR activation, whereas MT2A knockdown had minimal impact (Figure 3E; Figure S5A–C). Consistent with these findings, depletion of MT1E, MT1F, or MT1M, but not MT2A, similarly sensitized AR‑activated VCaP cells to cuproptosis (Figure 3F; Figure S5D–F).

Consistently, depletion of MT1E, MT1F, or MT1M enhanced copper‐induced loss of FDX1 and Lip‐DLAT (Figure 3G–J) and significantly increased mitochondrial copper accumulation (Figure 3K–N). In contrast, MT2A knockdown did not appreciably affect copper distribution. Multiplex immunofluorescence (mIF) analysis of PDX#1 tumors further confirmed that AR inhibition reduced MT1E expression (Figure 3O). Together, these results demonstrate that AR suppresses cuproptosis through induction of MT1, which sequesters cytosolic copper, restricts its mitochondrial accumulation, and thereby buffers copper toxicity.

### 2.4 MTF1 Is a Critical Mediator Linking AR to MT1 Regulation

**FIGURE 4:**
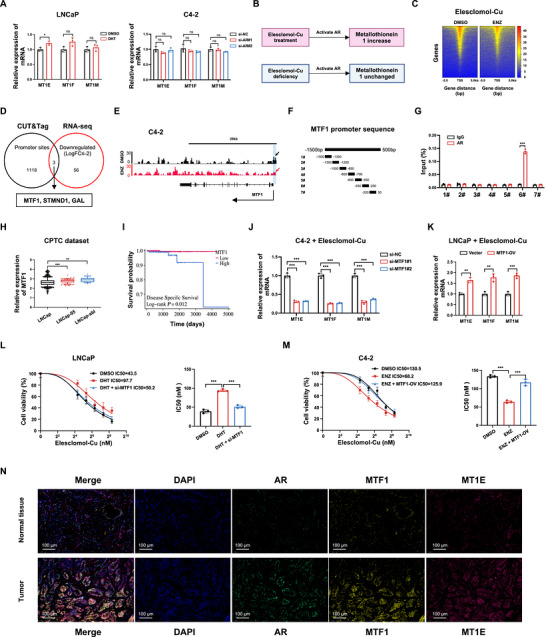
MTF1 is a critical mediator linking AR to MT1 regulation. (A) mRNA expression of MT1E, MT1F, and MT1M in AR‐activated LNCaP cells (Left) and in AR‐knockdown C4‐2 cells (Right). (B) Schematic diagram of AR regulation of MT1 with or without exogenous copper. (C) CUT&Tag density heatmap of AR enrichment in C4‐2 cells within ±5 kb around TSS. (D) Intersection between genes with significantly reduced AR promoter binding peaks upon ENZ treatment and genes significantly downregulated (LogFC ≤ −2) upon AR knockdown (from RNA‐seq in Figure 3A). (E) IGV visualization of AR enrichment peaks in the MTF1 promoter region from CUT&Tag. (F) Schematic diagram of truncated fragments of the MTF1 promoter region used for analysis. (G) ChIP‐qPCR validation of AR binding to the MTF1 promoter fragment. (H) MTF1 expression in LNCaP, LNCaP‐95 (CRPC), and LNCaP‐abl (CRPC) cells from the CPTC dataset. (I) Association between MTF1 expression and disease‐specific survival in the TCGA prostate cancer cohort analyzed by Kaplan‐Meier analysis. (J) mRNA expression of MT1E, MT1F, and MT1M in MTF1‐knockdown C4‐2 cells (treated with Ele–Cu 100 nm, 12h). (K) mRNA expression of MT1E, MT1F, and MT1M in MTF1‐overexpressing LNCaP cells (treated with Ele–Cu 100 nm, 12h). (L) Relative cell viability of AR‐activated LNCaP cells with additional MTF1 knockdown, treated with Ele–Cu for 48 h. (m) Relative cell viability of AR‐inhibited C4‐2 cells with MTF1 overexpression, treated with Ele–Cu for 48 h. (N) Multiplex immunofluorescence of AR, MTF1, and MT1E in prostate cancer tissue and adjacent normal tissue. Scale bar 100 µm. All graphic data are presented as mean ± SD (*n* = 3 replicates) from one representative of three independent experiments. Statistical analysis by Student's *t*‐test (A left, G, K), one‐way ANOVA followed by Tukey's multiple comparisons test (A right, H, J, L, M). ns: no significance, ^*^ *p* < 0.05, ^**^ *p* < 0.01, ^***^ *p* < 0.001.

Although AR activity was correlated with MT1 family induction under copper stress, direct AR activation or inhibition failed to alter MT1 expression in the absence of exogenous copper (Figure 4A). This indicates that AR does not directly regulate MT1 transcription. These observations pointed to the existence of a copper‐responsive mediator linking AR to MT1 activation (Figure 4B). To identify such a mediator, we integrated CUT&Tag profiling performed in C4‐2 cells under Ele–Cu treatment (Figure 4C; Figure S6A) with RNA‐seq data. This intersection yielded three candidate genes (Figure 4D). Among them, MTF1 uniquely displayed a strong positive correlation with AR expression, whereas STMND1 and GAL showed no such relationship (Figure S6B). IGV visualization further revealed diminished AR occupancy at the MTF1 promoter following ENZ treatment (Figure 4E). Consistent with this, ChIP‐seq analysis in the absence of Ele–Cu treatment also showed AR enrichment at the MTF1 promoter region, with a significant reduction upon AR inhibition (Figure S6C). Concordantly, AR activation increased MTF1 expression, while AR inhibition markedly suppressed it (Figure S6D–G). In silico analysis using the JASPAR database [32] identified putative AR‐binding motifs within the MTF1 promoter (Figure S6H), and ChIP–qPCR confirmed AR enrichment at the sixth promoter fragment (Figure 4F,G; Figure S6I). This fragment contains a putative AR‑binding motif. To test whether this motif mediates AR‑dependent activation, we generated a luciferase reporter carrying point mutations in the predicted binding sequence (Figure S6J). DHT significantly induced the wild‑type MTF1 promoter activity, whereas this induction was abolished in the mutant reporter, confirming that AR directly activates MTF1 transcription through this motif (Figure S6K). Supporting these findings, CPTC data showed elevated MTF1 expression in high–AR activity cell lines (LNCaP‐95 and LNCaP‐abl) relative to parental LNCaP cells (Figure 4H). Clinically, high MTF1 expression was associated with poor prognosis in prostate cancer patients (Figure 4I).

To determine whether MTF1 functionally mediates MT1 induction under copper stress, we generated MTF1 knockdown and overexpression models (Figure S7A,B). Under copper stress, MTF1 depletion markedly suppressed, whereas MTF1 overexpression enhanced, MT1 expression (Figure 4J, K). Consistent with these transcriptional changes, loss of MTF1 impaired cell growth under copper stress, while MTF1 overexpression alleviated cuproptosis (Figure S7C,D). Importantly, MTF1 depletion abolished AR‐mediated resistance to cuproptosis (Figure 4L; Figure S7E), whereas MTF1 overexpression rescued AR‐inhibited cells from cuproptosis (Figure 4M; Figure S7F).

To further validate the role of MTF1 in mediating AR‑driven cuproptosis resistance, we overexpressed MTF1 in ENZ‑treated VCaP cells. As shown in Figure S7G, MTF1 overexpression significantly rescued AR‑inhibited VCaP cells from cuproptosis, consistent with the observations in C4‑2 cells. Mechanistically, MTF1 suppression reversed AR‐driven increases in MT1 expression and cytosolic copper retention, accompanied by restored mitochondrial copper accumulation (Figure S7H). Conversely, enforced MTF1 expression counteracted the effects of AR inhibition on MT1 expression and copper subcellular distribution (Figure S7I). These alterations were mirrored by changes in core cuproptosis markers: MTF1 depletion exacerbated FDX1 and Lip‐DLAT loss and promoted DLAT oligomerization, whereas MTF1 overexpression mitigated these effects (Figure S7J,K). Finally, mIF analysis of human prostate cancer specimens revealed coordinated activation of the AR–MTF1–MT1E axis in tumor regions with elevated copper content compared with adjacent normal tissues (Figure 4N). Together, these results identify MTF1 as a key mediator of tumor cell adaptation to copper stress and a central factor enabling AR‐driven resistance to cuproptosis.

### 2.5 EP300‐Mediated Lactylation of MTF1 at K218 Enhances Its Nuclear Translocation

**FIGURE 5:**
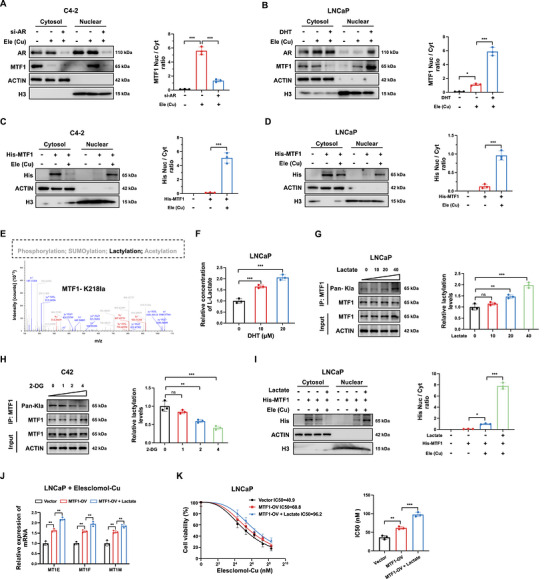
MTF1 undergoes lactylation in prostate cancer cells. (A, B) Nuclear and cytoplasmic distribution of AR and MTF1 in AR‐knockdown C4‐2 cells (A) and AR‐activated LNCaP cells (B), both treated with Ele–Cu (100 nm, 12 h). (C, D) Nuclear and cytoplasmic distribution of His‐MTF1 in C4‐2 (C) and LNCaP cells (D) treated with Ele–Cu (100 nm, 12 h). (E) Identification of MTF1 PTMs by LC‐MS/MS. (F) Lactate levels in LNCaP cells treated with DHT. (G) Lactylation levels of MTF1 in LNCaP cells treated with lactate (0–40 µm). (H) Lactylation levels of MTF1 in C4‐2 cells treated with 2‐DG (0–4 mm). (I) Nuclear and cytoplasmic distribution of His‐MTF1 in LNCaP cells treated with lactate (40 µm) and Ele–Cu (100 nm) for 12 h. (J) mRNA expression of MT1E, MT1F, and MT1M in MTF1‐overexpressing LNCaP cells treated with lactate (40 µm) and Ele–Cu (100 nm) for 12 h. (K) Relative cell viability of MTF1‐overexpressing LNCaP cells treated with lactate (40 µM) and Ele–Cu for 48 h. All graphic data are presented as mean ± SD (*n* = 3 replicates) from one representative of three independent experiments. Statistical analysis by Student's *t*‐test (C, D). Comparisons among multiple groups were performed by one‐way ANOVA followed by Tukey's multiple comparisons test (A, B, F, G, H, I, J, K). ns: no significance, ^*^ *p* < 0.05, ^**^ *p* < 0.01, ^***^ *p* < 0.001.

Nuclear translocation of MTF1 is required for activation of downstream metallothionein genes in response to copper stress. Consistent with this, MTF1 overexpression or knockdown did not alter MT1 expression in the absence of copper stimulation (Figure S8A,B). Under steady‐state conditions, MTF1 was predominantly localized in the cytoplasm and underwent nuclear translocation upon Ele–Cu stimulation. AR knockdown reduced total MTF1 abundance and was accompanied by decreased nuclear MTF1 under copper stress (Figure 5A); Conversely, DHT stimulation increased overall MTF1 expression and led to a marked enrichment of MTF1 in the nucleus following copper exposure, with cytoplasmic MTF1 being nearly entirely redistributed to the nuclear compartment (Figure 5B). These observations suggested that AR activity may influence not only MTF1 expression but also its nuclear translocation efficiency in response to copper stimulation. To further evaluate this possibility independent of endogenous expression levels, we ectopically expressed His‐tagged MTF1 in prostate cancer cells. Upon copper stimulation, only a fraction of exogenous His–MTF1 translocated to the nucleus in LNCaP cells, whereas nearly complete nuclear localization was observed in C4‐2 cells under identical conditions, supporting a role for high AR activity in enhancing MTF1 nuclear import efficiency (Figure 5C,D). Consistently, upon Ele–Cu treatment, His‑MTF1 showed increased nuclear accumulation in the presence of DHT (Figure S8C).

Given the critical role of PTMs such as phosphorylation, acetylation, lactylation, and SUMOylation in regulating protein nuclear translocation [33, 34, 35], we hypothesized that the differential nuclear translocation efficiency of MTF1 under high AR activity might be attributable to its PTM status. To identify regulatory PTMs, MTF1 was immunoprecipitated and subjected to liquid chromatography–mass spectrometry (LC–MS/MS) analysis for phosphorylation, acetylation, lactylation, and SUMOylation. Among these candidate modifications, lysine lactylation was uniquely detected, revealing a previously unrecognized lactylation site at 218 lysine residue (K218) (Figure 5E). Because lactate serves as the substrate for protein lactylation, we measured intracellular lactate levels and found significantly higher concentrations in CRPC cells (C4‐2, 22Rv1, and VCaP) compared with LNCaP cells (Figure S8D). DHT stimulation increased lactate production in LNCaP cells (Figure 5F). Exogenous lactate treatment enhanced MTF1 lactylation in LNCaP cells (Figure 5G), whereas inhibition of glycolysis with 2‐deoxyglucose (2‐DG) reduced MTF1 lactylation in C4‐2 cells (Figure 5H). These results suggest that AR‐dependent metabolic reprogramming modulates MTF1 lactylation. Functionally, lactate supplementation markedly enhanced MTF1 nuclear translocation in LNCaP cells under copper stress (Figure 5I). The increase in nuclear MTF1 was accompanied by upregulation of MT1 genes (Figure 5J) and increased resistance to cuproptosis (Figure 5K).

**FIGURE 6:**
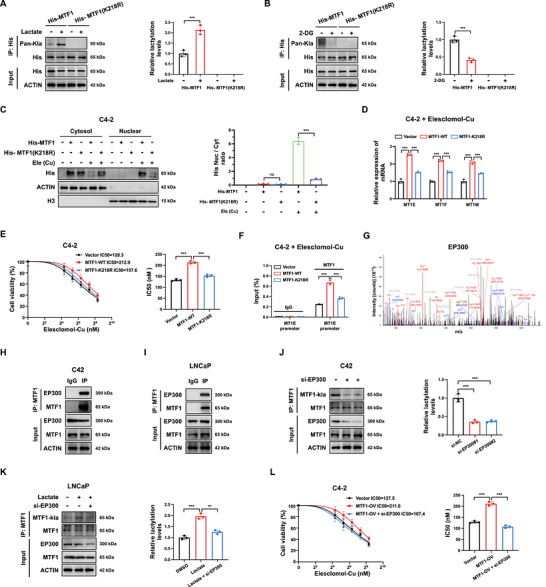
EP300‐mediated lactylation of MTF1 at lysine 218 enhances nuclear translocation. (A, B) A: MTF1 lactylation in LNCaP cells expressing His‐MTF1 or His‐MTF1‐K218R and treated with lactate (40 µm). B: MTF1 lactylation in C4‐2 cells expressing His‐MTF1 or His‐MTF1‐K218R and treated with 2‐DG (4 mm). (C) Nuclear and cytoplasmic distribution of His‐MTF1 or His‐MTF1‐K218R in C4‐2 cells treated with Ele–Cu (100 nm, 12 h). (D) mRNA expression of MT1E, MT1F, and MT1M in C4‐2 cells transfected with His‐MTF1 or His‐MTF1‐K218R and treated with Ele–Cu (100 nm, 12 h). (E) Relative viability of C4‐2 cells expressing His‐MTF1 or His‐MTF1‐K218R and treated with Ele–Cu for 48 h. (F) ChIP‑qPCR validation of MTF1 binding to the MT1E promoter in C4‐2 cells expressing His‐MTF1 or His‐MTF1‐K218R and treated with Ele–Cu (100 nm, 12 h). (G) LC‐MS/MS showing an interaction between MTF1 and EP300. (H, I) Co‐IP of MTF1 and EP300 in C4‐2 (H) and LNCaP (I) cells. (J) Lactylation levels of MTF1 in C4‐2 cells upon EP300 knockdown. (K) Lactylation levels of MTF1 in LNCaP cells with EP300 knockdown and lactate treatment (40 µm). (L) Relative cell viability of MTF1‐overexpressing C4‐2 cells with additional EP300 knockdown, treated with Ele–Cu for 48 h. All graphic data are presented as mean ± SD (*n* = 3 replicates) from one representative of three independent experiments. Statistical analysis by Student's *t*‐test (A, B). Comparisons among multiple groups were performed by one‐way ANOVA followed by Tukey's multiple comparisons test (C, D, E, F, J, K, L). ^**^ *p* < 0.01, ^***^ *p* < 0.001.

To determine whether lactylation occurs specifically at K218, we generated a lysine‐to‐arginine mutant (MTF1‐K218R). Lactate stimulation failed to induce lactylation of MTF1‐K218R (Figure 6A), and 2‐DG treatment did not further reduce its modification, confirming K218 as the principal lactylation site (Figure 6B). Compared with wild‐type MTF1, the K218R mutant exhibited impaired nuclear translocation upon copper stimulation, reduced induction of MT1 genes, and diminished tolerance to cuproptosis (Figure 6C–E). Because reduced nuclear localization corresponded with decreased MT1 transcription, we next assessed MTF1 promoter occupancy. ChIP–qPCR confirmed MTF1 binding to the promoters of MT1E, MT1F, and MT1M (Figure S9A–C), and this enrichment was markedly reduced in cells expressing the K218R mutant (Figure 6F; Figure S9D,E).

To identify the possible lysine lactyltransferase responsible for MTF1 lactylation, we performed immunoprecipitation (IP) of MTF1 followed by LC–MS/MS analysis of interacting proteins. The proteins specifically detected in the MTF1 IP group (excluding those present in the IgG control) are provided in Table S1. Among the candidates, EP300 was selected for further validation based on its known role as a lysine lactyltransferase (Figure 6G). Co‐IP confirmed the interaction between MTF1 and EP300 (Figure 6H,I). EP300 knockdown in C4‐2 cells markedly reduced MTF1 lactylation (Figure 6J), and similarly suppressed lactate‐induced MTF1 lactylation in LNCaP cells (Figure 6K). Functionally, depletion of EP300 reversed the increase in copper tolerance conferred by MTF1 overexpression in C4‐2 cells (Figure 6L). Collectively, these findings demonstrate that EP300‐mediated lactylation of MTF1 at lysine 218 promotes MTF1 nuclear translocation, enhances MT1 transcription, and strengthens cellular tolerance to copper.

### 2.6 ARv7 Drives MTF1‐Dependent Cuproptosis Resistance in ARPI‐Resistant CRPC

**FIGURE 7:**
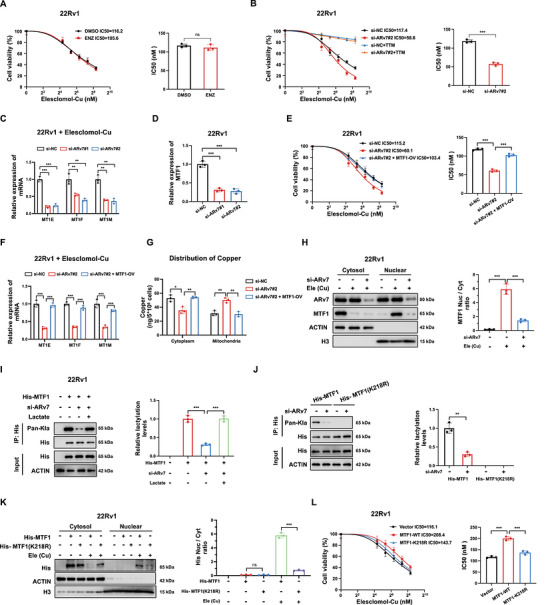
ARv7 suppresses cuproptosis through MTF1 in ARPI‑resistant CRPC. (A) Relative cell viability of ENZ‐treated 22Rv1 cells treated with Ele–Cu for 48 h. (B) Relative cell viability of ARv7‐knockdown 22Rv1 cells treated with Ele–Cu for 48 h, rescued by TTM (1 µm). (C) mRNA expression of MT1E, MT1F, and MT1M in ARv7‐knockdown 22Rv1 cells, treated with Ele–Cu 100 nm for 12h. (D) mRNA expression of MTF1 in ARv7‐knockdown 22Rv1 cells. (E) Relative cell viability of ARv7‐knockdown 22Rv1 cells with MTF1 overexpression treated with Ele–Cu for 48 h. (F) mRNA expression of MT1E, MT1F, and MT1M in ARv7‐knockdown 22Rv1 cells with MTF1 overexpression, treated with Ele–Cu 100 nm for 12h. (G) Mitochondrial and cytoplasmic copper content in ARv7‐knockdown 22Rv1 cells with MTF1 overexpression (treated with Ele–Cu 100 nm, 12 h). (H) Nuclear and cytoplasmic distribution of ARv7 and MTF1 in ARv7‐knockdown 22Rv1 cells (treated with Ele–Cu 100 nm, 12 h). (I) Lactylation levels of His‑MTF1 in 22Rv1 cells transfected with si‑ARv7, with or without lactate supplementation. (J) Lactylation levels of His‑MTF1 or His‑MTF1‑K218R in 22Rv1 cells transfected with si‑ARv7. (K) Nuclear and cytoplasmic distribution of His‐MTF1 or His‐MTF1‐K218R in 22Rv1 cells treated with Ele–Cu (100 nm, 12 h). (L) Relative viability of 22Rv1 cells expressing His‐MTF1 or His‐MTF1‐K218R and treated with Ele–Cu for 48 h. All graphic data are presented as mean ± SD (*n* = 3 replicates) from one representative of three independent experiments. Statistical analysis by Student's *t*‐test (A, B, J). Comparisons among multiple groups were performed by one‐way ANOVA followed by Tukey's multiple comparisons test (C, D, E, F, G, H, I, K, L). ns: no significance, ^*^ *p* < 0.05, ^**^ *p* < 0.01, ^***^ *p* < 0.001.

Long‐term treatment with ARPI frequently leads to the emergence of ARPI‐resistant CRPC. Among the underlying mechanisms, the ligand‑binding‑deficient AR splice variant 7 (ARv7) lacks the C‑terminal ligand‑binding domain (LBD), rendering it insensitive to ENZ or other ARPI that target the LBD. Unlike full‑length AR, ARv7 can drive downstream transcription independent of androgenic stimulation, thus representing a major driver of ARPI resistance [36, 37]. 22Rv1 cells exhibited strong resistance to ENZ (Figure S10A) and therefore served as a representative ARPI‐resistant CRPC model. Consistently, ENZ treatment or total AR knockdown did not significantly reduce KLK3 expression (Figure S10B–D). In 22Rv1 cells, AR inhibition failed to alter sensitivity to cuproptosis (Figure 7A; Figure S10E), suggesting that canonical AR signaling is not the dominant regulator of copper tolerance in this context. Because ARv7 is the principal driver in 22Rv1 cells, we next examined its role in copper stress adaptation. ARv7‐specific knockdown reduced KLK3 expression (Figure S10F–H) and markedly sensitized cells to cuproptosis (Figure 7B; Figure S10I).

Consistent with the effects observed upon AR inhibition in canonical AR‐driven CRPC models, ARv7 depletion increased DLAT oligomerization while reducing Lip‐DLAT and FDX1 protein levels (Figure S10J,K), suggesting that ARv7 similarly regulates mitochondrial copper distribution. RNA‐seq analysis further revealed enrichment of the mineral absorption pathway following ARv7 knockdown (Figure S10L,M). Under copper stress, ARv7 depletion markedly suppressed expression of MT1 (Figure 7C). Given that MT1 transcription is regulated by MTF1, we next examined whether ARv7 controls MT1 expression through MTF1. ChIP‐qPCR analysis in 22Rv1 cells, using the same MTF1 promoter primers as validated in Figure 4F, revealed that ARv7 is significantly enriched at the MTF1 promoter region compared with IgG control (Figure S10N). Indeed, ARv7 knockdown reduced MTF1 expression (Figure 7D; Figure S10O), and MTF1 depletion similarly decreased MT1 expression under exogenous copper treatment (Figure S10P). Functionally, MTF1 overexpression reversed the increased sensitivity to cuproptosis caused by ARv7 depletion (Figure 7E), restored MT1 expression (Figure 7F), and reduced mitochondrial copper accumulation (Figure 7G).

We next examined whether ARv7 also affects MTF1 nuclear translocation. Nuclear–cytoplasmic fractionation demonstrated that ARv7 depletion impaired MTF1 nuclear accumulation under copper stress (Figure 7H). Given the role of MTF1 lactylation in promoting its nuclear translocation, we hypothesized that ARv7 might influence MTF1 lactylation. Indeed, lactate levels were significantly reduced upon ARv7 knockdown (Figure S10Q). To exclude the confounding effect of ARv7 on endogenous MTF1 expression, we transfected 22Rv1 cells with His‑MTF1 and found that ARv7 knockdown decreased His‑MTF1 lactylation, which was rescued by lactate supplementation (Figure 7I). Furthermore, the MTF1‑K218R mutant abolished His‑MTF1 lactylation, indicating that ARv7 modulates MTF1‑K218 lactylation through regulation of intracellular lactate levels (Figure 7J). Consistently, mutation of the MTF1 lactylation site (K218R) compromised MTF1 nuclear translocation and cellular tolerance to cuproptosis in 22Rv1 cells (Figure 7K, L). Collectively, these findings demonstrate that ARv7 sustains resistance to cuproptosis through the MTF1‑dependent copper‑buffering program, highlighting MTF1 as a potential therapeutic target across distinct stages of CRPC.

### 2.7 Diphenylfulvene Targeting MTF1 Unleashes Cuproptosis to Suppress CRPC Growth

**FIGURE 8:**
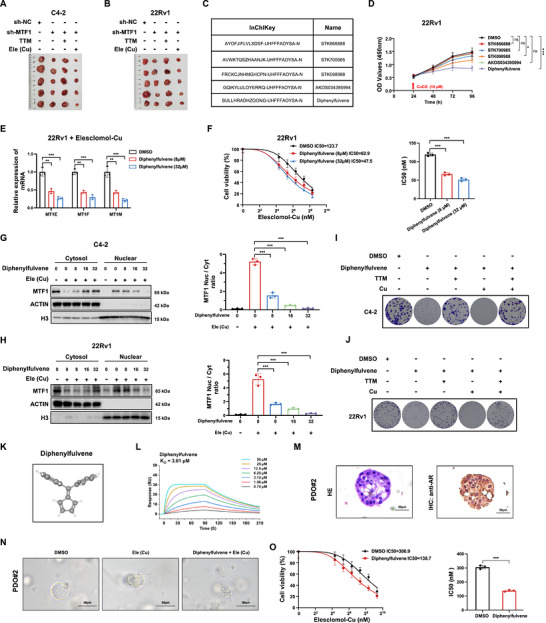
Targeting MTF1 induces cuproptosis in CRPC. (A) Representative images of MTF1‐knockdown C4‐2 xenografts treated with TTM (0.1 g/L in drinking water from day 1) or Ele–Cu (10 mg/kg, intratumoral injection, every 2 days starting from day 10) (*n* = 5 mice per group). (B) Representative images of MTF1‐knockdown 22Rv1 cell xenografts treated with TTM (0.1 g/L in drinking water from day 1) or Ele–Cu (10 mg/kg, intratumoral injection, every 2 days starting from day 10) (*n* = 5 mice per group). (C) Top 5 small molecule compounds selected after excluding drugs annotated with biological toxicity in PubChem. (D) Proliferation of 22Rv1 cells treated individually with five small molecule compounds (8 µm) in the presence of exogenous Cu (10 µm). (E) mRNA expression of MT1E, MT1F, and MT1M in Diphenylfulvene‐treated 22Rv1 cells (concurrently treated with Ele–Cu 100 nm, 12h). (F) Relative cell viability of 22Rv1 cells treated with Diphenylfulvene and Ele–Cu for 48 h. (G, H) Nuclear and cytoplasmic distribution of MTF1 in C42 (G) or 22Rv1(H) cells treated with a gradient of Diphenylfulvene (concurrent with Ele–Cu 100 nm, 12 h). (I, J) Representative images of a 14‐day colony formation assay in C42 (**I**) and 22Rv1 (J) cells treated with the MTF1 inhibitor Diphenylfulvene (32 µm) alone or in combination with TTM (1 µM) or low‐dose Cu (0.1 µm). (K) Structure of Diphenylfulvene from PubChem. (L) SPR kinetics fit for Diphenylfulvene binding to MTF1. (M) HE staining and anti‐AR immunohistochemistry (IHC) in PDO#2. Scale bar 50 µm. (N) Bright‐field images of PDO#2 after drug treatment for 48 h (scale bar 50 µm). (O)Relative cell viability of PDO#2 treated with Diphenylfulvene (32 µm) and Ele–Cu for 48 h. All graphic data are presented as mean ± SD (*n* = 3 replicates) from one representative of three independent experiments unless otherwise specified. Statistical analysis by Student's *t*‐test (O). Comparisons among multiple groups were performed by one‐way ANOVA followed by Tukey's multiple comparisons test (D, E, F, G, H). ns: no significance, ^*^ *p* < 0.05, ^**^ *p* < 0.01, ^***^ *p* < 0.001.

Given the critical role of MTF1 in sustaining AR‐driven copper accumulation in CRPC, we hypothesized that targeting MTF1 might disrupt copper homeostasis and trigger cuproptosis independent of exogenous copper supplementation. To test this, we established stable MTF1‐knockdown CRPC cell–derived xenograft (CDX) models (Figure S11A). In a C4‐2 cell–derived xenograft model, even in the absence of exogenous copper supplementation, MTF1 depletion markedly suppressed tumor growth. This inhibitory effect was largely reversed by the copper chelator TTM and further enhanced by low‐dose copper supplementation (Figure 8A; Figure S11B,C). Consistently, in a 22Rv1 CDX model, MTF1 knockdown also significantly inhibited tumor growth without exogenous copper supplementation (Figure 8B; Figure S11D,E). Then, mIF staining further revealed a pronounced reduction in MT1E expression following MTF1 depletion (Figure S11F). Collectively, these results demonstrate that loss of MTF1 alone is sufficient to disrupt endogenous copper homeostasis and trigger cuproptosis under basal copper conditions in CRPC, highlighting MTF1 as a central regulator of intrinsic copper buffering and a potential therapeutic vulnerability.

To identify potential MTF1 inhibitors, we performed virtual screening against a library of approximately 19 million small‑molecule compounds. Candidate compounds were ranked based on predicted binding affinity to MTF1 (residues 133–228). The top 50 candidates were listed in Table S2. After excluding compounds annotated with biological toxicity in the PubChem database, the top five ranked compounds were selected for further validation (Figure 8C). All five compounds displayed minimal cytotoxicity within the 2^1^–2^5^ µm concentration range (Figure S11G–K). Among them, Diphenylfulvene significantly reduced cell viability in the presence of exogenous copper (Figure 8D). Consistently, Diphenylfulvene suppressed MT1 expression and significantly potentiated cuproptosis (Figure 8E,F). We next sought to elucidate the mechanism by which Diphenylfulvene impairs MTF1 function. Diphenylfulvene treatment dose‐dependently inhibited copper‐induced nuclear translocation of MTF1 (Figure 8G,H), indicating that Diphenylfulvene blocks MTF1‐dependent transcription by preventing its nuclear import. As a consequence, mitochondrial copper buffering capacity mediated by the MT1 was compromised. Importantly, beyond sensitizing cells to copper ionophore–induced toxicity, Diphenylfulvene alone was sufficient to trigger cuproptosis in C4‐2 and 22Rv1 cells (Figure 8I,J), highlighting that restoration of endogenous copper‐induced cuproptosis represents a therapeutically exploitable vulnerability.

Molecular docking suggested stable binding between Diphenylfulvene and MTF1 (Figure 8K; Figure S11L). This interaction was further confirmed by surface plasmon resonance assays, which demonstrated direct binding between Diphenylfulvene and MTF1 (Figure 8L). To further evaluate its translational potential, we established PDO#2 from an individual with advanced cT4N1M1 prostate cancer (Figure 8M; Figure S11M). Diphenylfulvene significantly enhanced sensitivity to Ele–Cu in PDO#2 (Figure 8N,O). Together, these findings demonstrate that Diphenylfulvene inhibits MTF1 nuclear translocation, thereby disrupting MT1–mediated mitochondrial copper buffering and reactivating physiological cuproptosis, revealing a promising therapeutic strategy for advanced prostate cancer.

## 3 Discussion

The sensitivity of tumor cells to cuproptosis is determined not only by the abundance of FDX1 and lipoylated proteins [38, 39], but also by intracellular copper levels [40, 41, 42]. Here, we show that AR transcriptionally upregulates multiple STEAP metalloreductases, including STEAP1, STEAP2, and STEAP4, promoting extracellular Cu^2^^+^ reduction and facilitating copper import through SLC31A1. Because copper export via ATP7A and ATP7B remains largely unchanged, this imbalance results in net intracellular copper accumulation. These findings reconcile previously conflicting reports regarding androgen regulation of STEAP family members [43, 44, 45], and provide a mechanistic explanation for androgen‐stimulated copper uptake observed in earlier studies [24].

Despite elevated intracellular copper, AR activation does not increase copper toxicity but instead enhances tolerance. We resolve this paradox by identifying MT1 as the principal copper‐buffering system in prostate cancer. Metallothionein function appears highly context‐dependent across tumor types. While MT2A has been reported to suppress cuproptosis in colorectal and renal cancers under hypoxic or HIF1A‐activated conditions [46], our data identify MT1E, MT1F, and MT1M as the dominant copper‐chelating isoforms in prostate cancer. Considering the dynamic copper exchange between cytosol and mitochondria [47], we propose that the MT1 family acts via a dual mechanism: first, by binding free cytosolic copper to restrict its entry into mitochondria; second, by intercepting copper reflux from mitochondria to the cytosol, thereby limiting mitochondrial copper content on both fronts. Consistent with this model, we further show that AR activation does not directly alter the FDX1‐dependent lipoylation pathway but instead reduces mitochondrial copper availability, indirectly attenuating cuproptosis.

During our investigation of AR‐mediated regulation of MT1, we observed that AR failed to significantly induce MT1 expression in the absence of exogenous copper, whereas copper supplementation restored this effect. Given that AR transcriptional activity itself is not dependent on copper, we identified MTF1—an AR target gene—as the critical mediator linking AR to MT1 regulation. Structurally, MTF1 contains six N‐terminal Cys2His2 zinc fingers responsible for binding to metal response elements (MREs) and three C‐terminal transactivation domains that mediate transcriptional activation [48]. Previous work by Liu et al. demonstrated that SUMOylation at lysine 627 in the C‐terminus modulates MTF1 transcriptional activity without affecting its nuclear translocation or DNA‐binding ability [31]. In contrast, our study reveals that lysine 218, located within the MRE‐binding region, undergoes lactylation that markedly enhances MTF1 nuclear translocation. Lindert et al. previously reported that an atypical nuclear localization signal within zinc fingers 1–3 (amino acids 137–228) enables rapid nuclear entry; our findings further underscore the importance of this region in regulating MTF1 nuclear import [25]. Notably, lactylation‐dependent control of nuclear translocation has been reported in other contexts—for example, ABCF1‐K430 lactylation promotes its nuclear localization and binding to the KDM3A promoter, thereby enhancing transcription [33]. In CRPC, hyperactive AR drives glycolytic reprogramming [49, 50], generating abundant intracellular lactate to support protein lactylation. As an essential cellular metal, copper requires a relatively high threshold to trigger efficient MTF1 nuclear translocation [28]. Our data suggest that lactylation of MTF1 in CRPC enhances its nuclear import and effectively lowers the copper threshold required for activation, thereby strengthening cellular copper tolerance and protecting CRPC cells from copper‐induced toxicity.

The role of MTF1 in suppressing cuproptosis has been reported previously in gastric cancer, where MTF1 promotes assembly of Fe–S cluster proteins through ISCA2 to alleviate copper toxicity [51]. Our study establishes that copper‐driven MTF1 activation also operates in prostate cancer and is co‐opted by AR signaling to sustain copper tolerance. Importantly, we demonstrate that depletion of MTF1 under basal copper conditions—without exogenous copper or ionophores—significantly impairs tumor growth, an effect that can be reversed by the copper chelator TTM. These findings provide direct experimental evidence that physiological cuproptosis could occur once intrinsic copper buffering is disrupted.

In terms of translational application, our study employed multiple preclinical models. In both PDX and PDO models, combining ARPI with Ele significantly enhanced therapeutic efficacy. Consistent with our results, a recent study by Gao et al. showed that ENZ sensitizes murine prostate organoids to Ele–Cu, further supporting the translational potential of this combination strategy [52]. However, for patients with ARPI‐resistant CRPC, conventional therapies such as chemotherapy or PARP inhibition achieve limited benefit, with a median overall survival of only 12–15 months [53, 54]. To address this pressing clinical challenge, we propose disrupting copper homeostasis in advanced prostate cancer cells by pharmacologically targeting MTF1 to trigger cuproptosis. We identified Diphenylfulvene, a small‐molecule inhibitor of MTF1, through virtual screening, and demonstrated that it blocks MTF1 nuclear translocation, suppresses metallothionein expression, disrupts copper homeostasis, and reactivates cuproptosis in resistant models. Importantly, this compound triggers cuproptosis without the need for exogenous copper supplementation, underscoring its ability to harness intrinsic copper for therapeutic benefit.

**FIGURE 9:**
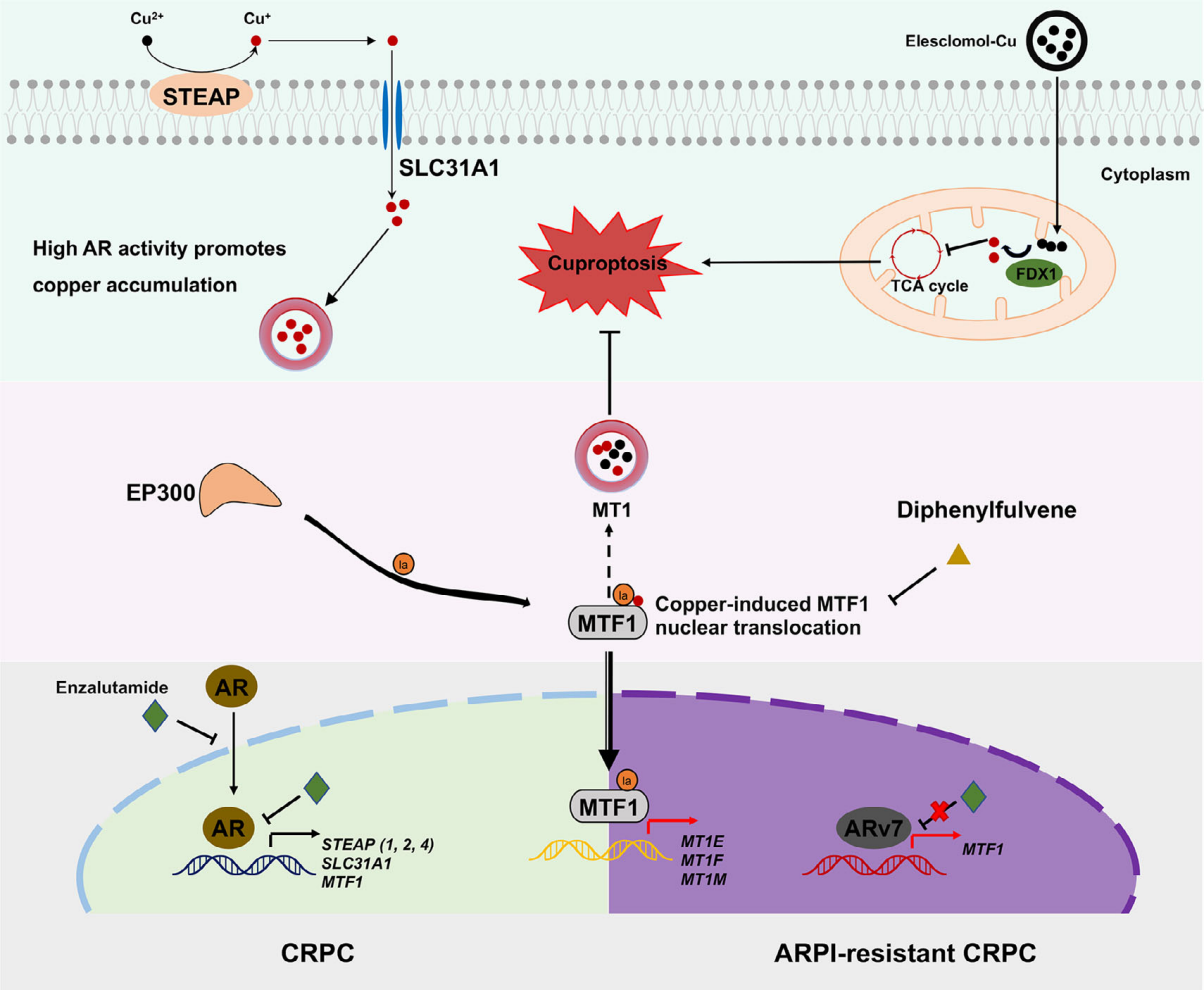
Schematic model.

In summary, AR signaling drives intracellular copper accumulation in prostate cancer, creating a cuproptosis‐permissive state that is buffered by the MTF1–MT1 axis. Targeting MTF1 disrupts this buffering system, unleashes cuproptosis, and bypasses both AR and ARv7 signaling (Figure 9). These findings define a mechanism underlying copper homeostasis in AR‐hyperactivated prostate cancer and highlight MTF1 as a therapeutic target to exploit endogenous copper for treating CRPC.

## 4 Materials and Methods

### 4.1 Human Studies

The prostate cancer cohort (PCa cohort) was established using patient samples collected at The Second Affiliated Hospital of Nanjing Medical University between July 2022 and July 2024. Exclusion criteria included prior androgen deprivation therapy or radiotherapy before surgery, as well as a history of additional malignant diagnoses. Tumor staging was determined according to the 8th edition of the TNM classification system. All patients received standard postoperative adjuvant therapy according to established protocols.

### 4.2 Cell Lines

The human prostate cancer cell lines LNCaP, VCaP, 22Rv1, PC‐3, and DU145 were obtained from the Type Culture Collection of the Chinese Academy of Sciences (China). The C4‐2 cell line was acquired from the American Type Culture Collection (ATCC, USA). LNCaP, C4‐2, and 22Rv1 cells were maintained in RPMI 1640 medium; VCaP and DU145 cells were cultured in DMEM; and PC‐3 cells were grown in F‐12K medium. All media were supplemented with 10% fetal bovine serum (FBS, Wisent, Canada) and incubated at 37°C in a 5% CO_2_ atmosphere. The following compounds were used: Enzalutamide (HY‐70002, MedChemExpress, USA), Dihydrotestosterone (S4757, Selleckchem, USA), Elesclomol (S1052, Selleckchem, USA), CuCl2 (751944, Sigma‐Aldrich, Germany), and Tetrathiomolybdate (HY‐128530, MedChemExpress, USA).

### 4.3 Analysis of Spatial Transcriptomic and Single‐Cell RNA Sequencing

Spatial transcriptomics (ST) and single‐cell RNA sequencing (scRNA‐seq) data from prostate cancer samples analyzed in this study are available in the OMIX database under accession number OMIX008930. Detailed methodologies regarding gene targets for malignant/benign tissue annotation in ST‐seq and cell type identification in scRNA‐seq have been described previously [55]. To assess AR activity, we utilized an AR target gene set (including KLK3 and KLK2) derived from the “ANDROGEN_RECEPTOR_NETWORK_IN_PROSTATE_CANCER” pathway in the Molecular Signatures Database (MSigDB) [56]. AR activity scores were computed using the “AddModuleScore” function in Seurat, with regions or cells in the top 50% of scores defined as high AR activity and the bottom 50% as low activity. Copper accumulation was quantified using a gene set from the “COPPER_ION_TRANSPORT” pathway in MSigDB. A copper accumulation score was calculated as the difference between the acquisition module (STEAP1, STEAP2, STEAP3, STEAP4, SLC31A1) and the export module (ATP7A, ATP7B), both evaluated with “AddModuleScore”. Cell clustering was performed at a resolution of 0.7 and visualized via Uniform Manifold Approximation and Projection (UMAP). The association between AR activity scores and copper accumulation scores was assessed using Spearman correlation analysis.

### 4.4 Copper Microplate Assay

Copper levels in prostate cancer tissues were quantified using a Copper Microplate Assay Kit (abs580140, Absin, China) following the manufacturer's instructions. Tissue lysates from 36 patients (Table S3) were homogenized in assay buffer at a ratio not exceeding 0.1 g tissue per 1 mL. Absorbance was measured at 605 nm, and copper content was normalized to tissue weight.

### 4.5 RNA Isolation and Quantitative Reverse Transcription‑Polymerase Chain Reaction (qRT‑PCR) and RNA Sequencing

Total RNA was extracted from cell lines and clinical tissue specimens using TRIzol reagent (Invitrogen, USA). cDNA was synthesized with HiScript II Q RT SuperMix (Vazyme, China) according to the manufacturer's instructions. qRT‑PCR was carried out on a LightCycler 480 system (Roche, USA) using ChamQ SYBR qPCR Master Mix (Vazyme, China). Sequences of all primers used are provided in Table S4. Total RNA was isolated from prostate cancer cells and control cells with TRIzol reagent (Invitrogen, USA), and RNA sequencing was performed by Majorbio (Shanghai, China).

### 4.6 Western Blot Analysis

Samples were lysed in RIPA buffer (NCM Biotech, China) supplemented with protease inhibitors (Sigma‐Aldrich, USA). Proteins were separated by SDS‐PAGE and electrotransferred to PVDF membranes (Millipore, USA). After incubation with primary antibodies overnight at 4°C, membranes were incubated with species‐matched HRP‐conjugated secondary antibodies for 1 h at room temperature. Protein signals were visualized using a chemiluminescence system (Bio‐Rad, USA). All experiments were independently repeated three times. The following primary antibodies were used: AR, ARv7, DLAT, ACTIN (1:1000, Cell Signaling Technology, USA); SLC31A1, STEAP1 (1:1000, Origene, USA); Lipoic acid, FDX1, Histone H3 (1:1000, Abcam, UK); MTF1, EP300 (1:1000, Proteintech, China).

### 4.7 Cell Transfection

Lentiviral constructs for AR overexpression, MTF1 overexpression, and MTF1 knockdown were purchased from Corues Technology (Nanjing, China). Small interfering RNAs (siRNAs) targeting AR, ARv7, MT1E, MT1F, MT1M, and MT2A were obtained from HANBIO (Shanghai, China) and transfected into prostate cancer cells using the Lipofectamine 3000 kit (Invitrogen, USA), following the manufacturer's protocol. The shRNAs and siRNAs used in this study were listed in Table S5.

### 4.8 Measurement of Cell Viability

For cell viability assessment, cells were plated in 96‐well plates at a density of 100 µL medium per well and allowed to adhere overnight. Following treatment with specified concentrations of Ele, Cu, or TTM for 48 h, 10 µL of Cell Counting Kit‐8 reagent (Dojindo Molecular Technologies, Japan) was added to each well. After incubating for 2 h, absorbance was measured at 450 nm. Half‐maximal inhibitory concentration (IC_50_) values were derived using GraphPad Prism software.

### 4.9 Clonogenic Survival Assay

Cells were seeded in 24‐well culture plates, and the medium was replaced every other day with fresh medium containing the indicated compounds or vehicle control. After 48 h, cells were fixed using 0.5% crystal violet solution (Beyotime, China).

### 4.10 Nuclear and Cytoplasmic Fractionation

Nuclear and cytoplasmic proteins were extracted using the NE‐PER Nuclear and Cytoplasmic Extraction Reagents (Thermo Fisher Scientific, USA) according to the manufacturer's instructions, with all procedures carried out on ice. Briefly, cells were harvested and washed with ice‐cold PBS. The cell pellet was resuspended in ice‐cold Cytoplasmic Extraction Reagent I (CER I), vortexed vigorously for 15 s, and incubated on ice for 10 min. Ice‐cold CER II was then added, followed by vortexing for 5 seconds and incubation on ice for 1 min. After centrifugation at 16,000 g for 5 min at 4°C, the supernatant (cytoplasmic extract) was collected. The insoluble pellet (nuclear fraction) was resuspended in ice‐cold Nuclear Extraction Reagent, vortexed for 15 sec, and incubated on ice with intermittent vortexing every 10 min for 40 min. The nuclear extract was centrifuged at 16,000 g for 10 min at 4°C, and the supernatant (nuclear extract) was collected. Extraction efficiency of the nucleus was validated by Western Blotting using Histone H3 antibody (Abcam).

### 4.11 Assessment of Mitochondrial Copper

Mitochondria were extracted using a Cell Mitochondria Isolation kit (C3601, Beyotime, China) according to the manufacturer's instructions. Briefly, cells were gently resuspended in PBS pre‐chilled on ice and centrifuged at 600 g for 5 min at 4°C to pellet the cells. The supernatant was discarded. Then, 1 mL of mitochondria isolation reagent was added, and the mixture was incubated on ice for 15 min. The cell suspension was transferred to a glass homogenizer and homogenized with 30 strokes. The homogenate was centrifuged at 1000 g for 10 min at 4°C. The supernatant was carefully transferred to another centrifuge tube and centrifuged at 11 000 g for 10 min at 4°C. The resulting supernatant represents the cytoplasmic fraction, and the pellet constitutes the mitochondrial fraction. Subsequently, the isolated mitochondria were lysed with 1 mL of mitochondrial lysis buffer. The homogenized lysates (0.5 mL) were precisely transferred into a transparent polytetrafluoroethylene centrifuge tube, followed by the addition of 5 mL of guaranteed reagent‐grade concentrated nitric acid. The mixture was vigorously vortexed for 1 min and then subjected to digestion in a 120°C heating block until the volume was reduced to approximately 1 mL and the digestate became clear and transparent. The resulting digestate was diluted to a final volume of 5 mL with ultrapure water, mixed by vortexing for 1 min, and filtered through a 0.22 µm sterile membrane filter to remove particulate impurities. The filtered sample was appropriately diluted based on expected concentration and analyzed for copper using an Inductively coupled plasma‐Mass Spectrometer (PE NexION 300D, PerkinElmer, USA).

### 4.12 High‐Throughput CUT&Tag

The CUT&Tag assay was performed using the Hyperactive Universal CUT&Tag Assay Kit for Illumina Pro (TD‐904, Vazyme Biotech Co., Ltd, China). Briefly, 50 000 cells were washed with Wash Buffer at room temperature and resuspended in 100 µL of the same buffer. The cell suspension was then incubated with activated ConA Beads Pro for 10 min at room temperature. After incubation, the supernatant was removed using a magnetic stand. The cell‐bead complexes were incubated overnight at 4°C with 50 µL of chilled Antibody Buffer containing 1 µL of AR antibody (Cell Signaling Technology). The following day, the supernatant was discarded. Secondary antibody (Vazyme, China), diluted 1:100 in Dig‐Wash Buffer, was added and incubated with rotation for 60 min at room temperature. After removal of the supernatant, the samples were washed with Dig‐Wash Buffer. The cell‐bead complexes were then incubated with diluted pA/G‐Tnp Pro for 1 h at room temperature with rotation. Subsequently, the samples were washed with Dig‐300 Buffer, and the supernatant was discarded. The complexes were resuspended in TTBL mixture and incubated at 37°C for 60 min in an open‐lid PCR thermal cycler. Then, 2 µL of 10% SDS and 0.5 pg of DNA Spike‐in were added, followed by incubation at 55°C for 10 min. The magnetic beads were discarded, and the supernatant was transferred to a new tube. The supernatant was incubated with activated DNA Extract Beads Pro for 20 min at room temperature. After discarding the supernatant, the beads were washed twice with 1 × B&W Buffer for 30 s each at room temperature. The beads were air‐dried briefly with the tube lid open at room temperature and then resuspended in 15 µL of ddH_2_O. Library amplification was performed using the TruePrep Index Kit V2 for Illumina (TD202, Vazyme) with 12 cycles under recommended thermal conditions. Then, 100 µL of VAHTS DNA Clean Beads (N411, Vazyme) were added to the product and incubated for 5 min at room temperature. The beads were washed twice with 80% ethanol, air‐dried for 5 min with lids open, and finally eluted in 22 µL of ddH_2_O. The resulting libraries were subjected to high‐throughput sequencing at Lianchuan Biological Information Co., Ltd (China). Sequencing data analysis was conducted on the Vazyme Cloud Platform (http://cloud.vazyme.com:83/).

### 4.13 Chromatin Immunoprecipitation (ChIP) Assay

A ChIP assay was conducted using the Magna ChIP Kit (17‐10085, Millipore, Germany) following the manufacturer's protocol. Chromatin was subjected to immunoprecipitation with anti‐AR antibody (4 µg, Cell Signaling Technology), anti‐AR‐V7 antibody (4 µg, Cell Signaling Technology), or anti‐MTF1 antibody (4 µg, Proteintech), using normal rabbit IgG as a negative control. The immunoprecipitated chromatin was then analyzed by qRT–PCR targeting genomic regions spanning from −1,500 bp to +500 bp relative to the transcription start site of target genes. Primer sequences targeting the promoter regions are provided in Table S6.

### 4.14 Luciferase Reporter Assay

To assess the interaction between AR and the MTF1 promoter, luciferase reporter plasmids containing the wild‑type (WT) or motif‑mutated (MT) MTF1 promoter region were obtained from GenePharma (China). LNCaP cells were co‑transfected with WT or MT reporter plasmids using Lipofectamine 3000 (Invitrogen, USA). After transfection, cells were treated with DHT (10 µm) or DMSO for 24 h. Firefly luciferase activities were then measured using the Dual‑Luciferase Reporter Assay System (Promega, USA) according to the manufacturer's instructions.

### 4.15 Measurement of Lactate

Lactate content was quantified using the LA Content Assay Kit (Solarbio, BC2235). Lactate was extracted from equal amounts of cells across different groups with extraction solutions A and B. According to the manufacturer's instructions, the extracted lactate was mixed with a color‐developing solution and transferred into a 96‐well plate. The lactate concentration was then determined by measuring the absorbance at 570 nm.

### 4.16 LC–MS/MS Analysis of MTF1 Post‐Translational Modifications

Cell lysates prepared in IP lysis buffer (Beyotime, Shanghai, China) were incubated with anti‐MTF1 antibody (1:100, Proteintech, China) or control IgG antibody (1:100, Proteintech, China) on a rotating platform at 4°C overnight. Protein A/G agarose beads (Thermo Scientific, USA) were then added and incubated with rotation at 4°C for an additional 6 h. The beads were washed three times with wash buffer to remove nonspecific binding. Bound proteins were eluted by adding 1 × SDS‐PAGE loading buffer and heating at 100°C for 10 min. The eluted samples were separated by SDS‐PAGE and visualized by silver staining. Gel bands corresponding to immunoprecipitated MTF1 were excised and subjected to LC–MS/MS analysis performed by OE Biotechnology (Shanghai, China) to identify post‐translational modification sites on MTF1.

### 4.17 Establishment of Patient‐Derived Organoid

Prostate cancer tissues (detailed patient information is listed in Table S7) were repeatedly washed until the supernatant cleared, then minced into 0.5–1 mm^3^ fragments using Tissue Dissociation Medium I (AimingMed, China). The fragments were digested in a shaking water bath for 60 min. Upon microscopic confirmation of cell cluster release, the digestion was quenched with washing solution and centrifuged. The pellet was further digested in Tissue Dissociation Medium II (AimingMed, China) for 10–15 min, filtered through a 100 µm strainer, and treated with Red Blood Cell Lysis Buffer (AimingMed, China). The resulting cells were resuspended in Prostate Cancer Organoid Complete Medium (AimingMed, China) supplemented with Primary Enhancer (AimingMed, China), mixed with Basement Membrane Matrix (AimingMed, China), and seeded as 50 µL domes in a 24‐well plate. After polymerization, 600 µL of pre‐warmed organoid medium was added. Organoids were cultured at 37°C under 5% CO_2_, with medium changes every 3 days and passaging every 8–12 days based on confluency.

### 4.18 Development of Patient‐Derived Xenograft Model

Tumor samples were transported on ice immediately after resection and macro‐dissected upon arrival in the laboratory. The clinical characteristics of the patients are summarized in Table S7. Fragments (approximately 50 mm^3^) were subcutaneously implanted into both scapular regions of six‐week‐old male NOG mice (Charles River Co., China) under isoflurane anesthesia, with carprofen administered for analgesia. This founding generation was designated as F1. PDX models were maintained through serial transplantation into new recipients when tumor volumes reached approximately 1500 mm^3^. For the F3 generation, ENZ treatment was initiated on day 12 at a daily oral dose of 10 mg/kg. Ele–Cu (1:1 molar ratio, 10 mg/kg) was administered via intratumoral injection every three days starting from day 15. Tumor dimensions were measured every three days using calipers, and volume was calculated as V = (length × width^2^)/2. All mice were euthanized one month after treatment initiation for subsequent analysis.

### 4.19 Xenograft Tumor Models

Male BALB/c nude mice (4 weeks old, Charles River Co., China) were subcutaneously injected in the axillary region with C4‐2 or 22Rv1 cells stably expressing either a control vector (sh‐NC) or an MTF1‐targeting shRNA (sh‐MTF1). The mice were then divided into groups receiving the following treatments: the sh‐NC group remained untreated; the sh‐MTF1 groups were further divided into no treatment, TTM (0.1 g/L in drinking water from day 1), or Ele‐Cu complex (1:1, 10 mg/kg, intraperitoneal injection every two days for five doses starting on day 10). Tumor dimensions and volume measurements were performed as previously described. After three weeks, all mice were euthanized, and tumors were harvested, weighed, fixed, and processed for further analysis.

### 4.20 Hematoxylin‐Eosin (HE) Staining and Immunohistochemistry (IHC)

For HE staining, tissue samples were fixed in 4% formalin, paraffin‐embedded, and sectioned at 4 µm. Sections were dewaxed in xylene, rehydrated through a graded ethanol series, stained with hematoxylin and eosin, dehydrated, cleared in xylene, and mounted with Permount mounting medium (Fisher Scientific).

For IHC, serial 4 µm sections were subjected to deparaffinization, rehydration, and antigen retrieval via microwave treatment. After inhibition of endogenous peroxidase activity and blocking with BSA, sections were incubated with primary antibodies at 4°C overnight. HRP‐conjugated secondary antibodies were then applied, followed by DAB development and hematoxylin counterstaining.

### 4.21 Multiplex Immunofluorescence (mIF)

mIF was performed using a five‐color TSA‐based fluorescence kit (Recordbio Biological Technology, Shanghai, China) according to the manufacturer's protocol. Formalin‐fixed paraffin‐embedded sections (4 µm) were deparaffinized in xylene, rehydrated through graded ethanol, and subjected to antigen retrieval in retrieval buffer by microwave treatment (medium power for 8 min, cooling for 8 min, and medium‐low power for 7 min). After washing with PBS (pH 7.4), endogenous peroxidase activity was quenched with 3% H_2_O_2_ for 15 min at room temperature in the dark. Sections were blocked with 3% BSA for 30 min before incubation with primary antibodies (diluted in antibody buffer) overnight at 4°C in a humidified chamber. Following PBS washes, HRP‐conjugated secondary antibodies were applied for 50 min at room temperature, and TSA fluorophores were developed for 10 min. This staining cycle was repeated for each antibody/fluorophore pair. Finally, nuclei were counterstained with DAPI for 10 min, and slides were mounted with anti‐fade mounting medium.

### 4.22 Virtual Screening of Small‐Molecule Compounds Targeting MTF1

We improved LigUnity for virtual screening [57]. PyMOL was used for visualization. Since the structure of the human MTF1 protein (UniProt ID: Q14872) and its binding pocket are unknown, we constructed a sequence‐based version of LigUnity by replacing the original pocket encoder with the sequence encoder ESM2 [58]. The modified model was trained on PocketAffDB [57]. For the virtual screening stage, the amino acid sequence region spanning residues 133–228 of MTF1 was used as the input to the sequence encoder. Screening was then performed on a compound library of approximately 19 million small molecules. The top 50 ranked candidate inhibitors were presented in Table S2.

### 4.23 Surface Plasmon Resonance (SPR)

The binding affinity between MTF1 protein and compound Diphenylfulvene was determined using SPR on a Biacore 1K system at 25°C. The target protein was immobilized onto Series S Sensor Chip CM5 via amine coupling. PBST was used as the running buffer. Serially diluted Diphenylfulvene samples (50 µm to 0.78 µm, 2‐fold dilution) were injected for binding analysis. All data were collected and analyzed with Biacore Insight Evaluation Software. Both the steady‐state affinity model and kinetic model were used for fitting and affinity calculation.

### 4.24 Publicly Available Data for Validation

The RNA‐seq and ChIP‐seq data used for validation were obtained from public repositories including TCGA and the Gene Expression Omnibus (GEO). The dataset GSE279661 was employed to examine gene expression changes following AR activation. Additionally, ChIP‐seq data from GSE252897 were utilized to validate AR binding at promoter regions of target genes.

### 4.25 Statistical Analysis

All experiments were independently repeated at least three times. Data are expressed as mean ± standard deviation (SD) and were analyzed using GraphPad Prism (version 10, GraphPad Software, La Jolla, CA, USA). Comparisons between two groups were performed using Student's *t*‐test, while one‐way ANOVA followed by Tukey's multiple comparisons test was applied for multi‐group comparisons. A *p*‐value of less than 0.05 was considered statistically significant.

## Author Contributions

Conceptualization: K. L., B. Y., Q. Z., and M. D.; Methodology: Y. S. L., M. W., J. X., M. Y., A. S., R. Y., and Z. F.; Investigation: K. L., Y. W., F. L., Y. J. L., Y. D., J. L., and M. Y.; Visualization: J. W., Y. Z., Y. L., T. Z., and K. Z.; Funding acquisition: Q. Z., J. L.; Project administration: M. D., M. G., B. Y., and Q. Z.; Supervision: B. Y., M. D., M. G., and Q. Z.; Writing – original draft: K. L., Y. S. L., and J. L.; Writing – review & editing: B. Y., M. D., and Q. Z.

## Funding

This study is supported by the Scientific Research Project of Jiangsu Provincial Health Commission grant ZD2021028 (Q. Z.). Scientific Research Project of Jiangsu Provincial Health Commission grant YJYC200506 (Q. Z.). National Natural Science Foundation of China grant 82503824 (J. L.).

## Ethics Statement

Animal studies were approved by the Animal Research Ethics Committee of Nanjing Medical University (Approval No. 2406024), and all animal procedures were carried out in accordance with institutional animal welfare regulations. Patients in this study were included with informed consent and institutional ethical approval (2023‐KY‐050‐01).

## Conflicts of Interest

The authors declare no conflict of interest.

## Supporting information




**Supporting File 1**: advs77110‐sup‐0001‐SuppMat.docx.

**Supporting File 2**: advs77110‐sup‐0002‐Data.zip.

## Data Availability

The data that support the findings of this study are available from the corresponding author upon reasonable request.
