# Supplementary material for: EP300‐Mediated MTF1‐K218 Lactylation Buffers AR‐Driven Copper Overload to Suppress Cuproptosis in Castration‐Resistant Prostate Cancer
Source: Adv Sci (Weinh). 2026 Aug 11;13(61):e77110. doi: 10.1002/advs.77110 (PMC13460415; doi:10.1002/advs.77110)
Supplement: Supplementary file 1 [file ADVS-13-e77110-s001.docx]

**Supplementary Figure**

**Figure S1. Correlation between AR activity and copper accumulation in ST-seq and scRNA-seq of prostate cancer.**

(**A**) mRNA expression of KLK3 in AR-activated LNCaP cells treated with DHT (10 μM).

(**B**) Relative cell viability of C4-2 cells treated with ENZ for 48h.

(**C**) mRNA expression of KLK3 in AR-inhibited C4-2 cells treated with ENZ (10 μM).

(**D**) Standard curve for copper detection by ICP-MS.

(**E**) Dot plot of marker gene expression for each cell type from scRNA-seq, with dot size representing the percentage of cells expressing the gene and color representing the average scaled expression value.

(**F**) UMAP analysis showing the distribution of high and low AR activity and copper accumulation scores within epithelial cells in scRNA-seq.

(**G**) Association between AR activity score and copper accumulation score analyzed by Spearman's correlation analysis in scRNA-seq.

(**H**) UMAP analysis of scRNA-seq data from epithelial cells showing benign and malignant clustering in scRNA-seq.

(**I**) Dot plot showing the marker gene expression for each cluster, where the dot size represents the percentage of cells expressing the gene and the color represents the average scaled expression value in scRNA-seq.

(**J**) Dot plot of marker gene expression for tumor and normal regions from ST-seq.

All graphic data are presented as mean ± SD (n = 3 replicates) from one representative of three independent experiments unless otherwise specified. Statistical analysis by Student’s t test (**A**, **C**). ***p < 0.001.

**Figure S2. AR promotes the expression of copper uptake genes.**

(**A**) Protein levels of STEAP1 and SLC31A1 in AR-activated LNCaP cells (DHT 10μM).

(**B**) Protein levels of STEAP1 and SLC31A1 in AR-inhibited C4-2 cells (ENZ 10μM).

(**C**) ChIP-qPCR validation of AR binding to the STEAP1 promoter fragment.

(**D**) ChIP-qPCR validation of AR binding to the STEAP2 promoter fragment.

(**E**) ChIP-qPCR validation of AR binding to the STEAP4 promoter fragment.

(**F**) ChIP-qPCR validation of AR binding to the SLC31A1 promoter fragment.

(**G–J**) IGV visualization of AR ChIP‑seq peaks at the promoter regions of STEAP1 (**G**), STEAP2 (**H**), STEAP4 (**I**), and SLC31A1 (**J**). Data source: GSE252897.

All graphic data are presented as mean ± SD (n = 3 replicates) from one representative of three independent experiments. Statistical analysis by Student’s t test (**A–F**). *p < 0.05, **p < 0.01, ***p < 0.001.

**Figure S3. AR inhibits cuproptosis in prostate cancer.**

(**A, B**) Relative cell viability of LNCaP cells (**A**) and C4-2 cells (**B**) treated with Cu for 48h, rescued by concurrent treatment with TTM (1 mM).

(**C**) Representative images of clonogenic survival assay using DHT-activated LNCaP cells treated with Ele–Cu (100 nM) for 48 h, followed by rescue with TTM (1 μM).

(**D, E**) AR overexpression efficiency in LNCaP cells.

(**F**) mRNA expression of KLK3 in AR-overexpressing LNCaP cells with or without DHT activation (10 μM).

(**G**) Relative cell viability of DHT-activated, AR-overexpressing LNCaP cells treated with Ele–Cu for 48h.

(**H**) Representative images of AR-inhibited C4-2 cells treated with Ele–Cu (150 nM) for 48h, rescued by TTM (1 μM).

(**I, J**) AR knockdown efficiency in C4-2 cells.

(**K**) mRNA expression of KLK3 in AR-knockdown C4-2 cells.

(**L**) Relative cell viability of AR-knockdown C4-2 cells treated with Ele–Cu for 48h, rescued by TTM (1 μM).

(**M**) mRNA expression of KLK3 in AR-inhibited VCaP cells treated with ENZ (10 μM).

(**N**) Workflow for generating patient-derived organoids from prostate cancer.

(**O**) MRI images (T2-weighted and DWI) of the patient for PDO#1.

(**P**) Establishment of a PDX model.

(**Q**) MRI images (T2-weighted and DWI) of the patient for PDX#1.

All graphic data are presented as mean ± SD (n = 3 replicates) from one representative of three independent experiments. Statistical analysis by Student’s t test (**D, E, L, M**), comparisons among multiple groups were performed by one-way ANOVA followed by Tukey’s multiple comparisons test (**F, G, J, K**). **p < 0.01, ***p < 0.001.

**Figure S4. AR does not directly affect FDX1 or DLAT.**

(**A, B**) Protein levels of DLAT oligomers, Lip-DLAT, and FDX1 in AR-activated LNCaP cells (**A**, DHT, 10 μM) and AR-knockdown C4-2 cells (**B**), both treated with Ele–Cu (100 nM, 12 h).

(**C, D**) mRNA expression of FDX1 and DLAT in AR-activated LNCaP cells (**C**, DHT, 10 μM) and AR-knockdown C4-2 cells (**D**), both treated with Ele–Cu (100 nM, 12 h).

(**E, F**) Protein expression of Lip-DLAT and FDX1 in AR-activated LNCaP cells (**E**, DHT, 10 μM) and in AR-knockdown C4-2 cells (**F**).

(**G, H**) mRNA expression of FDX1 and DLAT in AR-activated LNCaP cells (**G**, DHT, 10 μM) and in AR-knockdown C4-2 cells (**H**).

(**I, J**) Expression of FDX1 and Lip-DLAT in LNCaP (**I**) and C4-2 cells (**J**), both treated with Ele–Cu (0-150 nM, 12h).

All graphic data are presented as mean ± SD (n = 3 replicates) from one representative of three independent experiments. Statistical analysis by Student’s t test (**A, C, E, G**), comparisons among multiple groups were performed by one-way ANOVA followed by Tukey’s multiple comparisons test (**B, D, F, H, I, J**). ns: no significance, *p < 0.05, **p < 0.01, ***p < 0.001.

**Figure S5. MT1 family members, but not MT2A, mediate AR‑mediated resistance to cuproptosis.**

(**A–C**) Relative cell viability of AR-activated LNCaP cells following knockdown of MT1F (**A**), MT1M (**B**), or MT2A (**C**) and treated with Ele–Cu for 48h.

(**D–F**) Relative cell viability of AR-activated VCaP cells following knockdown of MT1F (**D**), MT1M (**E**), or MT2A (**F**) and treated with Ele–Cu for 48h.

All graphic data are presented as mean ± SD (n = 3 replicates) from one representative of three independent experiments. Comparisons among multiple groups were performed by one-way ANOVA followed by Tukey’s multiple comparisons test (**A–F**). ns: no significance, **p < 0.01, ***p < 0.001.

**Figure S6. MTF1 is a target gene of AR.**

(**A**) AR CUT&Tag signal height and position relative to transcription start sites (TSS) for all genes in C4-2 cells treated with 100 nM Ele–Cu for 12h.

(**B**) Correlation analysis between AR and STMND1, GAL or MTF1 expression using TCGA prostate cancer data.

(**C**) IGV visualization of AR ChIP‑seq peaks at the promoter regions of MTF1. Data source: GSE252897.

(**D, E**) mRNA expression of MTF1 in AR-knockdown C4-2 cells (**D**) and in AR-activated LNCaP cells (**E,** DHT, 10 μM).

(**F, G**) Protein level of MTF1 in AR-knockdown C4-2 cells (**F**) and AR-activated LNCaP cells (**G**).

(**H**) Prediction of MTF1 binding sites using JASPAR.

(**I**) Agarose gel electrophoresis validating ChIP-qPCR products.

(**J**) Sequence alignment of the wild‑type (WT) and mutant (MUT) AR‑binding motifs within the MTF1 promoter.

(**K**) Relative luciferase activity of LNCaP cells transfected with WT or MUT MTF1 promoter reporter plasmids and treated with DHT (10 μM) or DMSO for 24 h.

All graphic data are presented as mean ± SD (n = 3 replicates) from one representative of three independent experiments. Statistical analysis by Student’s t test (**E, G, K**). Comparisons among multiple groups were performed by one-way ANOVA followed by Tukey’s multiple comparisons test (**D**, **F**). ns: no significance, ***p < 0.001.

**Figure S7.** **MTF1 suppresses cuproptosis.**

(**A, B**) Protein level of MTF1 in MTF1-knockdown C4-2 cells (**A**) and MTF1-overexpressing LNCaP cells (**B**).

(**C, D**) Relative cell viability of MTF1-knockdown C4-2 cells (**C**) and MTF1-overexpressing LNCaP cells (**D**) treated with Ele–Cu for 48h.

(**E**) Representative images of DHT-activated, MTF1-knockdown LNCaP cells treated with Ele–Cu (100 nM) for 48h, rescued by TTM (1 μM).

(**F**) Representative images of ENZ-inhibited, MTF1-overexpressing C4-2 cells treated with Ele–Cu (150 nM) for 48h, rescued by TTM (1 μM).

(**G**) Relative cell viability of AR-inhibited VCaP cells with MTF1 overexpression, treated with Ele–Cu for 48h.

(**H**) mRNA expression of MT1E, MT1F, and MT1M in AR-activated LNCaP cells with MTF1 knockdown (Left, treated with Ele–Cu 100 nM, 12h). Mitochondrial and cytoplasmic copper content in AR-activated LNCaP cells with MTF1 knockdown (Right, treated with Ele–Cu 100 nM, 12h).

(**I**) mRNA expression of MT1E, MT1F, and MT1M in AR-inhibited C4-2 cells with MTF1 overexpression (Left, treated with Ele–Cu 100 nM, 12h). Mitochondrial and cytoplasmic copper content in AR-inhibited C4-2 cells with MTF1 overexpression (Right, treated with Ele–Cu 100 nM, 12h).

(**J**) Protein levels of DLAT oligomers, Lip-DLAT, and FDX1 in AR-activated LNCaP cells with MTF1 knockdown (treated with Ele–Cu 100 nM, 12h).

(**K**) Protein levels of DLAT oligomers, Lip-DLAT, and FDX1 in AR-inhibited C4-2 cells with MTF1 overexpression (treated with Ele–Cu 100 nM, 12h).

All graphic data are presented as mean ± SD (n = 3 replicates) from one representative of three independent experiments. Statistical analysis by Student’s t test (**B, D**). Comparisons among multiple groups were performed by one-way ANOVA followed by Tukey’s multiple comparisons test (**A**, **C**, **G, H, I, J, K**). **p < 0.01, ***p < 0.001.

**Figure S8.** **AR promotes MTF1 nuclear translocation.**

(**A**) mRNA expression of MT1E, MT1F, and MT1M in MTF1-overexpression C4-2 cells.

(**B**) mRNA expression of MT1E, MT1F, and MT1M in MTF1-knockdown C4-2 cells.

(**C**) Confocal microscopy images of His‑MTF1 localization in LNCaP cells treated with DMSO, Ele–Cu, or Ele–Cu plus DHT. Scale bar, 2.5 μm.

(**D**) Lactate levels in prostate cancer cells.

All graphic data are presented as mean ± SD (n = 3 replicates) from one representative of three independent experiments. Statistical analysis by Student’s t test (**A**). Comparisons among multiple groups were performed by one-way ANOVA followed by Tukey’s multiple comparisons test (**B–D**). ns: no significance, *p < 0.05, ***p < 0.001.

**Figure S9. MTF1 binds to promoters of MT1 genes (MT1E, MT1F, MT1M).**

(**A**) ChIP-qPCR validation of MTF1 binding to the MT1E promoter fragment.

(**B**) ChIP-qPCR validation of MTF1 binding to the MT1F promoter fragment.

(**C**) ChIP-qPCR validation of MTF1 binding to the MT1M promoter fragment.

(**D**) ChIP‑qPCR validation of MTF1 binding to the MT1F promoter in C4-2 cells expressing His-MTF1 or His-MTF1-K218R and treated with Ele–Cu (100 nM, 12 h).

(**E**) ChIP‑qPCR validation of MTF1 binding to the MT1M promoter in C4-2 cells expressing His-MTF1 or His-MTF1-K218R and treated with Ele–Cu (100 nM, 12 h).

All graphic data are presented as mean ± SD (n = 3 replicates) from one representative of three independent experiments. Statistical analysis by Student’s t test (**A**–**C**). Comparisons among multiple groups were performed by one-way ANOVA followed by Tukey’s multiple comparisons test (**D**, **E**). **p < 0.01, ***p < 0.001.

**Figure S10. ARv7 inhibits cuproptosis in 22Rv1.**

(**A**) Relative cell viability of 22Rv1 cells treated with ENZ for 48h.

(**B**) mRNA expression of KLK3 in 22Rv1 cells treated with ENZ (50 μM).

(**C**) AR knockdown efficiency in 22Rv1 cells.

(**D**) mRNA expression of KLK3 in AR-knockdown 22Rv1 cells.

(**E**) Relative cell viability of AR-knockdown 22Rv1 cells treated with Ele–Cu for 48h.

(**F, G**) ARv7 knockdown efficiency in 22Rv1 cells.

(**H**) mRNA expression of KLK3 in ARv7-knockdown 22Rv1 cells.

(**I**) Representative images of ARv7-knockdown 22Rv1 cells treated with Ele-Cu (150 nM) for 48 h, rescued by TTM (1 μM).

(**J, K**) Protein levels of DLAT oligomers, Lip-DLAT, and FDX1 in ARv7-knockdown 22Rv1 cells (treated with Ele–Cu 100 nM, 12h).

(**L, M**) Volcano plot (**L**) and KEGG pathway enrichment analysis (**M**) from RNA-seq of ARv7-knockdown 22Rv1 cells treated with Ele–Cu (100 nM, 12h).

(**N**) ChIP-qPCR validation of ARv7 binding to the MTF1 promoter fragment.

(**O**) Protein level of MTF1 in ARv7-knockdown 22Rv1 cells.

(**P**) mRNA expression of MT1E, MT1F, and MT1M in MTF1-knockdown 22Rv1 cells (treated with Ele–Cu 100 nM, 12 h).

(**Q**) Lactate levels in 22Rv1 cells transfected with si‑ARv7 or si‑NC.

All graphic data are presented as mean ± SD (n = 3 replicates) from one representative of three independent experiments. Statistical analysis by Student’s t test (**B, N**). Comparisons among multiple groups (**C**, **D**, **E, F**, **G, H,** **K**, **O, P, Q**) were performed by one-way ANOVA followed by Tukey’s multiple comparisons test. ns: no significance, **p < 0.01, ***p < 0.001.

**Figure S11. Targeting MTF1 elicits physiological cuproptosis in CRPC.**

(**A**) Schematic of the drug administration regimen in xenografts.

(**B and C**) Tumor weight (**B**), and growth curve (**C**) of xenografts derived from MTF1-knockdown C4-2 cells, treated with either TTM or Ele–Cu. TTM was administered in drinking water (0.1 g/L) from day 1; Ele–Cu was delivered via intratumoral injection (10 mg/kg) every 2 days starting from day 10 (n=5 mice per group).

(**D** and **E**) Tumor weight (**D**), and growth curve (**E**) of xenografts derived from MTF1-knockdown 22Rv1 cells, treated with either TTM or Ele–Cu. TTM was administered in drinking water (0.1 g/L) from day 1; Ele–Cu was delivered via intratumoral injection (10 mg/kg) every 2 days starting from day 10 (n=5 mice per group).

(**F**) mIF of AR, MTF1, and MT1E in C4-2 xenograft tumor tissue. Scale bar 100 μm.

(**G–K**) Cell viability of 22Rv1 cells treated with increasing concentrations of five small molecule compounds for 48 h.

(**L**) Molecular docking model of Diphenylfulvene with MTF1.

(**M**) MRI images (T2-weighted and DWI) of the patient for PDO#2.

All graphic data are presented as mean ± SD (n = 3 replicates) from one representative of three independent experiments unless otherwise specified. Comparisons among multiple groups were performed by one-way ANOVA followed by Tukey’s multiple comparisons test (**B**, **C**, **D**, **E**). ns: no significance, *p < 0.05, **p < 0.01, ***p < 0.001.

**Supplementary Tables**

**Table S1. MTF1-interacting proteins identified by IP–MS after exclusion of IgG control**

| **Accession** | **Gene Symbol** | **Coverage [%]** | **# Peptides** | **# Unique Peptides** |
| --- | --- | --- | --- | --- |
| P68363 | TUBA1B | 33 | 11 | 11 |
| Q14872 | MTF1 | 35 | 13 | 13 |
| P07437 | TUBB | 43 | 14 | 4 |
| P68371 | TUBB4B | 46 | 15 | 5 |
| P10809 | HSPD1 | 35 | 16 | 16 |
| P25705 | ATP5F1A | 29 | 11 | 11 |
| P06576 | ATP5F1B | 30 | 12 | 12 |
| P35579 | MYH9 | 9 | 14 | 13 |
| P60709 | ACTB | 32 | 9 | 9 |
| P61978 | HNRNPK | 25 | 7 | 7 |
| P14618 | PKM | 21 | 8 | 8 |
| P30101 | PDIA3 | 19 | 6 | 6 |
| P68104 | EEF1A1 | 17 | 6 | 6 |
| Q09472 | EP300 | 23 | 35 | 31 |
| P68871 | HBB | 46 | 6 | 6 |
| P06744 | GPI | 13 | 5 | 5 |
| P69905 | HBA1; HBA2 | 49 | 4 | 4 |
| P06733 | ENO1 | 10 | 4 | 4 |
| P34897 | SHMT2 | 12 | 4 | 4 |
| P02768 | ALB | 12 | 6 | 6 |
| P08238 | HSP90AB1 | 7 | 4 | 4 |
| P31943 | HNRNPH1 | 7 | 2 | 2 |
| P78371 | CCT2 | 10 | 3 | 3 |
| P27797 | CALR | 11 | 3 | 3 |
| P51648 | ALDH3A2 | 9 | 4 | 4 |
| P16989 | YBX3 | 8 | 2 | 2 |
| Q7Z406 | MYH14 | 1 | 2 | 1 |
| P53597 | SUCLG1 | 8 | 2 | 2 |
| P36957 | DLST | 7 | 3 | 3 |
| Q86YZ3 | HRNR | 4 | 2 | 2 |
| Q15084 | PDIA6 | 7 | 2 | 2 |
| P36578 | RPL4 | 5 | 2 | 2 |
| P07237 | P4HB | 6 | 3 | 3 |
| P50990 | CCT8 | 5 | 2 | 2 |
| Q5D862 | FLG2 | 1 | 2 | 2 |
| P01857 | IGHG1 | 8 | 2 | 2 |
| Q9UN86 | G3BP2 | 5 | 2 | 2 |
| P17987 | TCP1 | 4 | 2 | 2 |
| P48643 | CCT5 | 5 | 2 | 2 |
| P50991 | CCT4 | 2 | 1 | 1 |
| Q13423 | NNT | 2 | 2 | 2 |
| P12268 | IMPDH2 | 2 | 1 | 1 |
| Q8NC51 | SERBP1 | 7 | 2 | 2 |
| P19474 | TRIM21 | 6 | 2 | 2 |
| P31025 | LCN1 | 13 | 2 | 2 |
| O43175 | PHGDH | 4 | 2 | 2 |
| P01861 | IGHG4 | 9 | 2 | 2 |
| P81605 | DCD | 10 | 1 | 1 |
| P13804 | ETFA | 6 | 1 | 1 |
| P62191 | PSMC1 | 3 | 1 | 1 |
| P62987 | UBA52 | 13 | 1 | 1 |
| O14556 | GAPDHS | 3 | 1 | 1 |
| Q13283 | G3BP1 | 2 | 1 | 1 |
| Q969S9 | GFM2 | 2 | 1 | 1 |
| P05455 | SSB | 3 | 1 | 1 |
| P09622 | DLD | 2 | 1 | 1 |
| P00390 | GSR | 2 | 1 | 1 |
| P55809 | OXCT1 | 2 | 1 | 1 |
| P40227 | CCT6A | 4 | 1 | 1 |
| O76031 | CLPX | 2 | 1 | 1 |
| Q96HU8 | DIRAS2 | 6 | 1 | 1 |
| Q8IVF7 | FMNL3 | 1 | 1 | 1 |
| P38646 | HSPA9 | 2 | 1 | 1 |
| Q9Y623 | MYH4 | 2 | 1 | 1 |
| P14868 | DARS1 | 2 | 1 | 1 |
| O00148 | DDX39A | 2 | 1 | 1 |
| Q02790 | FKBP4 | 2 | 1 | 1 |
| P48444 | ARCN1 | 2 | 1 | 1 |
| O60701 | UGDH | 2 | 1 | 1 |
| P35637 | FUS | 3 | 1 | 1 |
| Q8IZP2 | ST13P4 | 4 | 1 | 1 |
| P02763 | ORM1 | 3 | 1 | 1 |
| P22492 | H1-6 | 11 | 1 | 1 |
| P16402 | H1-3 | 5 | 1 | 1 |
| P26599 | PTBP1 | 2 | 1 | 1 |
| Q9Y6N5 | SQOR | 4 | 1 | 1 |
| O94925 | GLS | 2 | 1 | 1 |
| Q92734 | TFG | 2 | 1 | 1 |
| P49748 | ACADVL | 2 | 1 | 1 |
| P14866 | HNRNPL | 2 | 1 | 1 |
| Q15233 | NONO | 2 | 1 | 1 |
| Q96T60 | PNKP | 2 | 1 | 1 |
| Q9H254 | SPTBN4 | 1 | 1 | 1 |
| Q15046 | KARS1 | 1 | 1 | 1 |

**Table S2: Top 50 candidate inhibitors of MTF1**

| InChIKey | Value |
| --- | --- |
| AYOFJIFLVLXDSF-UHFFFAOYSA-N | 0.5864 |
| AVWKTQSZHAANJK-UHFFFAOYSA-N | 0.5664 |
| FRCKCJNHMGHCPN-UHFFFAOYSA-N | 0.5415 |
| CLGYYBCBWAMVSR-UHFFFAOYSA-N | 0.54 |
| WPDAVTSOEQEGMS-UHFFFAOYSA-N | 0.538 |
| GQIKYLULOYERRQ-UHFFFAOYSA-N | 0.536 |
| LFAYMJXHGYUQNV-UHFFFAOYSA-N | 0.536 |
| GVJFFQYXVOJXFI-UHFFFAOYSA-N | 0.536 |
| MWPLVEDNUUSJAV-UHFFFAOYSA-N | 0.535 |
| BULLHRADHZGONG-UHFFFAOYSA-N | 0.5337 |
| MBSOGQPCBUKDPG-UHFFFAOYSA-N | 0.532 |
| XUXUZNXCDVFRDB-UHFFFAOYSA-N | 0.5312 |
| NYZDIQPEOJSBLZ-UHFFFAOYSA-N | 0.531 |
| WDECIBYCCFPHNR-UHFFFAOYSA-N | 0.5303 |
| ICHCZSRMJBBXSJ-UHFFFAOYSA-N | 0.5273 |
| QBNLPAPGLLSKBN-UHFFFAOYSA-N | 0.5254 |
| FFAVWFCMRWYSIG-UHFFFAOYSA-N | 0.5234 |
| CMZYGFLOKOQMKF-UHFFFAOYSA-N | 0.5205 |
| QVRDLHPBZKOROD-UHFFFAOYSA-N | 0.519 |
| HBFJJKFTOQAJHO-UHFFFAOYSA-N | 0.5186 |
| KDUIUFJBNGTBMD-BONZMOEMSA-N | 0.5186 |
| IMBNBIFROMWBTM-MDWZMJQESA-N | 0.5186 |
| VTUNKULRSRAFHV-SCGPFSFSSA-N | 0.517 |
| IZIWAKQMDZZQBA-UHFFFAOYSA-N | 0.516 |
| ASVDRLYVNFOSCI-UHFFFAOYSA-N | 0.516 |
| MWQZYLIXDPNHFG-UHFFFAOYSA-N | 0.516 |
| OGVRJXPGSVLDRD-UHFFFAOYSA-N | 0.514 |
| AAAQKTZKLRYKHR-UHFFFAOYSA-N | 0.514 |
| JHDDCNAKLSSZJV-UHFFFAOYSA-N | 0.514 |
| OMIBPZBOAJFEJS-UHFFFAOYSA-N | 0.514 |
| KCZWGFUQVGFUAN-UHFFFAOYSA-N | 0.5137 |
| ZHTROMYSDSTCCE-UHFFFAOYSA-N | 0.5137 |
| UZMWDTVBYPAEMH-UHFFFAOYSA-N | 0.513 |
| TURIHPLQSRVWHU-UHFFFAOYSA-N | 0.5127 |
| BISWTLZVOHMIIC-UHFFFAOYSA-N | 0.5127 |
| WMMQAMSBVKPUSP-UHFFFAOYSA-N | 0.5127 |
| DOWJXOHBNXRUOD-UHFFFAOYSA-N | 0.512 |
| FZXCWGXBQYVKFP-UHFFFAOYSA-N | 0.512 |
| GZAFWGTUUHYQDL-UHFFFAOYNA-N | 0.512 |
| ZMYIIHDQURVDRB-UHFFFAOYSA-N | 0.5117 |
| JSFHRGSTXHEVCE-UHFFFAOYSA-N | 0.511 |
| BBQLNZRCZBCVPI-UHFFFAOYSA-N | 0.511 |
| VBTIFVJQJIIFBC-UZDSLJDTSA-N | 0.5107 |
| FMMWHPNWAFZXNH-UHFFFAOYSA-N | 0.5107 |
| LKZDZJZLWQDVEI-UHFFFAOYSA-N | 0.5103 |
| GMCJDXQKUIREGJ-UDWIEESQSA-N | 0.5103 |
| KTHUKEZOIFYPEH-UHFFFAOYSA-N | 0.5103 |
| COPYYWXEMCVZRM-UHFFFAOYSA-N | 0.51 |
| XCAVGRBZSYCJPM-UHFFFAOYSA-N | 0.5093 |
| HAUPSBCXPXMEIY-UHFFFAOYSA-N | 0.509 |

**Table S3. Clinical data of PCa cohort for copper microplate assay**

| **Clinical Values** | **Number** | |
| --- | --- | --- |
| Age |  |  |
| <70 | 21 |  |
| ≥70 | 15 |  |
| Gender |  |  |
| Male | 36 |  |
| PSA (ng/ml) |  |  |
| <20 | 28 |  |
| ≥20 | 8 |  |
| Biopsy Gleason score |  |  |
| ≤6 | 4 |  |
| 7 | 24 |  |
| ≥8 | 8 |  |
| pT |  |  |
| pT2 | 27 |  |
| pT3 | 7 |  |
| pT4 | 2 |  |
| pN |  |  |
| N0 | 31 |  |
| N1 | 5 |  |
| pM |  |  |
| M0 | 33 |  |
| M1 | 3 |  |

**Table S4: All PCR primers used in this research**

| Primers |  | sequence |
| --- | --- | --- |
| STEAP1 | Forward  Reverse | 5’- CCCTTCTACTGGGCACAATACA -3’  5’- GCATGGCAGGAATAGTATGCTTT -3’ |
| STEAP2 | Forward  Reverse | 5’- CAAGCGCGACAACAGGTTATT -3’  5’- CAGAGAGTAAAGAGTCGTAGGGG -3’ |
| STEAP3 | Forward  Reverse | 5’- CTCCCCGGAGGTCATCTTTG -3’  5’- TCTTGCTCTGTAGGGTTGCTC -3’ |
| STEAP4 | Forward  Reverse | 5’- GGCTTTGGGAATACTTGGGTT -3’  5’- TGGACAAATCGGAACTCTCTCC -3’ |
| SLC31A1 | Forward | 5’- ATGGAACCATCCTTATGGAGACA -3’ |
|  | Reverse | 5’- GGAAGTAGCTTATGACCACCTGG -3’ |
| ATP7A | Forward | 5’- TGACCCTAAACTACAGACTCCAA -3’ |
|  | Reverse | 5’- CGCCGTAACAGTCAGAAACAA -3’ |
| ATP7B | Forward | 5’- GGCCGTCATCACTTATCAGCC -3’ |
|  | Reverse | 5’- GGGAGCCACTTTGCTCTTGA -3’ |
| MT1E | Forward  Reverse | 5’- TCAGGTTGGGAGGGAACTCAA -3’  5’- GAAAGCCTGGAGAGGGAATGA -3’ |
| MT1F | Forward  Reverse | 5’- CGCCGCTGGTGTCTCC -3’  5’- CCCAGACCATGGAGATGGC -3’ |
| MT1M | Forward  Reverse | 5’- ACCTCCTGCAAGAAGAGCTG -3’  5’- CAGCAGCTGCAGTTCTCCAA -3’ |
| MT2A | Forward  Reverse | 5’- GATCCCAACTGCTCCTGCG -3’  5’- AGGAGCAGCAGCTTTTCTTG -3’ |
| FDX1 | Forward | 5’- TTCAACCTGTCACCTCATCTTTG -3’ |
|  | Reverse | 5’- TGCCAGATCGAGCATGTCATT -3’ |
| DLAT | Forward  Reverse | 5’- CGGAACTCCACGAGTGACC -3’  5’- CCCCGCCATACCCTGTAGT -3’ |
| MTF1 | Forward | 5’- CACAGTCCAGACAACAACATCA -3’ |
|  | Reverse | 5’- GCACCAGTCCGTTTTTATCCAC -3’ |
| KLK3 | Forward | 5’- GTGTGTGGACCTCCATGTTATT -3’ |
|  | Reverse | 5’- CCACTCACCTTTCCCCTCAAG -3’ |
| AR | Forward | 5’- GACGACCAGATGGCTGTCATT -3’ |
|  | Reverse | 5’- GGGCGAAGTAGAGCATCCT -3’ |
| ARv7 | Forward | 5’-CACCATGGAAGTGCAGTTAGGGCTG  GGAAGGGTCTACCCT -3’ |
|  | Reverse | 5’- TCAGGGTCTGGTCATTTTGAGATGCTTG  CAATTGCC -3’ |
| β-Actin | Forward | 5’- CTCCATCCTGGCCTCGCTGT -3’ |
|  | Reverse | 5’- GCTGTCACCTTCACCGTTCC -3’ |

**Table S5: All siRNAs/shRNAs used in this research**

| **Gene** | **Sequences (5’-3’)** |
| --- | --- |
| MTF1 shRNA-1 (Top strand) | AATTCGGTTATGTGCAGCACATTACTCGAGTAATGTGCTGCACATAACCTTTTTTG |
| MTF1 shRNA-1 (Bottom strand) | GATCCAAAAAAGGTTATGTGCAGCACATTACTCGAGTAATGTGCTGCACATAACCG |
| MTF1 shRNA-2 (Top strand) | AATTCGAAAGGTCATGATAACAAACTCGAGTTTGTTATCATGACCTTTCTTTTTTG |
| MTF1 shRNA-2 (Bottom strand) | GATCCAAAAAAGAAAGGTCATGATAACAAACTCGAGTTTGTTATCATGACCTTTCG |
| AR siRNA-1 | CGUGCAGCCUAUUGCGAGAUU |
| AR siRNA-2 | ACGUUUACUUAUCUUAUGCTT |
| ARv7 siRNA-1 | GCAAUUGCAAGCAUCUCAAUU |
| ARv7 siRNA-2 | GTAGTTGTGAGTATCATGATT |
| MT1E siRNA-1 | ATAGAGCAGCCAGTTGCAG |
| MT1E siRNA-2 | TGACTGCTTGTTCGTCTCA |
| MT1F siRNA-1 | CUGUGAAAUAUGUGAGUGA |
| MT1F siRNA-2 | GUAAACAGAGAGACAUGUA |
| MT1M siRNA-1 | GGACGUUGGAGAACUGCAG |
| MT1M siRNA-2 | AAGGGACGUUGGAGAACUG |
| MT2A siRNA-1 | CCGGUUCCUGCAAAUGCAATT |
| MT2A siRNA-2 | CGCUCCCAGAUGUAAAGAATT |
| EP300 siRNA-1 | CCGGUGAACUCUCCUAUAATT |
| EP300 siRNA-2 | CUAGAGACACCUUGUAGUATT |

**Table S6: Primer sequences targeting the promoter regions**

| Primers |  | sequence |
| --- | --- | --- |
| STEAP1 |  |  |
| 1# | Forward | 5’- TTGAAGGCGGCATGCTAGTT -3’ |
|  | Reverse | 5’- CTTCCGCAGTGTTTGTGTCC -3’ |
| 2# | Forward | 5’- ATGGAAAATGTGGTTGCGCG -3’ |
|  | Reverse | 5’- CCTCCCTTCCTTGCCTTAGC -3’ |
| 3# | Forward | 5’- CTTCAGGACCGGCTGTTAGG -3’ |
|  | Reverse | 5’- CAAGCGATTCTCCTGCCTCA -3’ |
| 4# | Forward | 5’- TCAGACCCCATCCCAGACAT -3’ |
|  | Reverse | 5’- TCCTCTCAAATGCCGTCTGC -3’ |
| STEAP2 |  |  |
| 1# | Forward | 5’- TTCCACACCCTGACACATCG -3’ |
|  | Reverse | 5’- ATGTAACCCCTCCCCTACCG -3’ |
| 2# | Forward | 5’- CCCCCTCTCGTTGTTGTTGT -3’ |
|  | Reverse | 5’- CTCCCTGCGGATTACCAAGT -3’ |
| 3# | Forward | 5’- CAAAGGTGCTCTCAGGGGAT -3’ |
|  | Reverse | 5’- CGGATAGGAGGACCCTCACA -3’ |
| 4# | Forward | 5’- ATCTCTGCAGTCCAGGGTCT-3’ |
|  | Reverse | 5’- GATTCCCACCCCAACTCTCG-3’ |
| STEAP4 |  |  |
| 1# | Forward | 5’- CCGGGAAGCGGGGATTTTAA -3’ |
|  | Reverse | 5’- AAGAACCCATGGCTTCCAGG -3’ |
| 2# | Forward | 5’- ACCACAGCCCTGGTTGTTTA -3’ |
|  | Reverse | 5’- AAGACCCATGGCTGAGTGGA -3’ |
| 3# | Forward | 5’- GTTCCTCCCATGGAAAGGGG -3’ |
|  | Reverse | 5’- GCAGTCCAGGACAAGACACA -3’ |
| 4# | Forward | 5’- ACAGGCCTAGGCTAGATGGT-3’ |
|  | Reverse | 5’- GGCCTTCACACTCTCTCACT-3’ |
| SLC31A1 |  |  |
| 1# | Forward | 5’- AAGACGGGGTAGGAAGGGAA -3’ |
|  | Reverse | 5’- TGTTGGGCCATGTGGTTTCT -3’ |
| 2# | Forward | 5’- AGGGGCATATGTGGTGCATT -3’ |
|  | Reverse | 5’- CCATTGTGTTGTGCCAGTGG -3’ |
| 3# | Forward | 5’- GTGCTAGGTACCATGCTGCA -3’ |
|  | Reverse | 5’- CTTGGTGGGTCAGCTCTAGC -3’ |
| 4# | Forward | 5’- GGCAAACCCAGGGCTATCTT -3’ |
|  | Reverse | 5’- ATACCGGCGGATTGTTGGAG -3’ |
| MTF1 |  |  |
| 1# | Forward  Reverse | 5’- CTATTTGGGGAGGGTGGCTG -3’  5’- CCCCGGCCAATGATTAGTGT -3’ |
| 2# | Forward  Reverse | 5’- CACTAATCATTGGCCGGGGT -3’  5’- AGTGGAAGTCCGTTGTTCCC -3’ |
| 3# | Forward  Reverse | 5’- ACCAACCCTGCCCATAGTTG -3’  5’- AGCCCCACAAAACACCTTCA -3’ |
| 4# | Forward  Reverse | 5’- TGAAGGTGTTTTGTGGGGCT -3’  5’- CTCCCACTTCATCCTGGCTG -3’ |
| 5# | Forward | 5’- GGAGTGAACCCAGCTGCTAG -3’ |
|  | Reverse | 5’- TGACTTCTCTCCCTCCCCAG -3’ |
| 6# | Forward | 5’- GGTATTCTGCTCGGGGATGG -3’ |
|  | Reverse | 5’- GAAGTATGTCTCCGCCGCTT -3’ |
| 7# | Forward | 5’- GGGAGAGAGCGAGAGACAGA -3’ |
|  | Reverse | 5’- GATTGAAGGAGACCGGTGGG -3’ |
| MT1E |  |  |
| 1# | Forward  Reverse | 5’- TTGACGCCATTCTCCTGCTT -3’  5’- CCTGGGCAACATAGGGAGTG -3’ |
| 2# | Forward  Reverse | 5’- TCTCAGGCGAGGAGAATGGA -3’  5’- TGTGTTCAGAACATCCGCCA -3’ |
| 3# | Forward  Reverse | 5’- CTCGGGGAAACTGGGAAAGG -3’  5’- GGAAAGCAGAATGGAGCCCT -3’ |
| 4# | Forward  Reverse | 5’- CCCTGGAATCCCCATTTCCC -3’  5’- AACTGGGTGATGTGGAGCAG -3’ |
| MT1F |  |  |
| 1# | Forward  Reverse | 5’- ACTTCTGGCCTGCAGTGATC -3’  5’- ACGTGCTCATCGTCACAGAG -3’ |
| 2# | Forward  Reverse | 5’- CAGTGATCTCGCTTCTGGGG -3’  5’- TAGTGTGCAAAGGACAGGCC -3’ |
| 3# | Forward  Reverse | 5’- AAGGCCAGGATCTCCAGGTA -3’  5’- TTGATAGTCAGGGGAGGGGG -3’ |
| 4# | Forward  Reverse | 5’- CCCACTGCTTCTTCGCTTCT -3’  5’- CAGCCTTGAGCCTCAGTACC -3’ |
| MT1M |  |  |
| 1# | Forward  Reverse | 5’- CTGCCACCATGCCAGGATAA -3’  5’- GCTGGTCGTCACAGAGCTAA -3’ |
| 2# | Forward  Reverse | 5’- GTGTTGCTGTGTACGGAGGA -3’  5’- CGTTTTCAACTGGGCCTGTC -3’ |
| 3# | Forward  Reverse | 5’- GAGGAGGGTGGAAGGCAAAG -3’  5’- TGCGGCTGCTTTTCTACTCA -3’ |
| 4# | Forward  Reverse | 5’- CTCTGGGTTTGAGGAGGTCG -3’  5’- GAGATTGCCAGCCAGAACCT -3’ |

**Table S7. Clinical data of PCa tissues for PDO and PDX**

| **Clinical Values** | **PDX#1** | **PDO#1** | **PDO#2** |
| --- | --- | --- | --- |
| Age | 77 | 74 | 70 |
| Gender | Male | Male | Male |
| PSA (ng/ml) | 42.3 | 6.88 | 162.0 |
| Biopsy Gleason score | 4+3 (7) | 3+4 (7) | 5+5 (10) |
| cT | 2 | 2 | 4 |
| cN | 0 | 0 | 1 |
| cM | 0 | 0 | 1 |
