# Supplementary material for: EP300‐Mediated MTF1‐K218 Lactylation Buffers AR‐Driven Copper Overload to Suppress Cuproptosis in Castration‐Resistant Prostate Cancer
Source: Adv Sci (Weinh). 2026 Aug 11;13(61):e77110. doi: 10.1002/advs.77110 (PMC13460415; doi:10.1002/advs.77110)

Fig. 2H

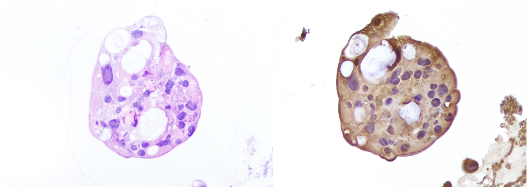

Fig. 2I

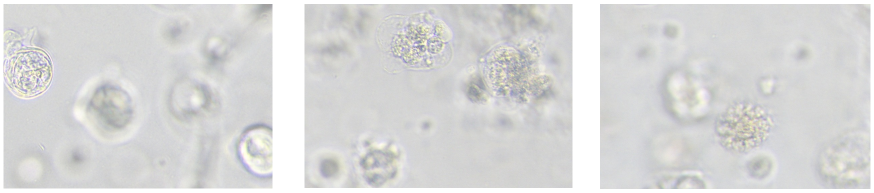

Fig. 2K

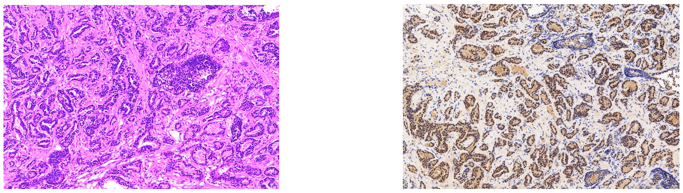

Fig. 3G

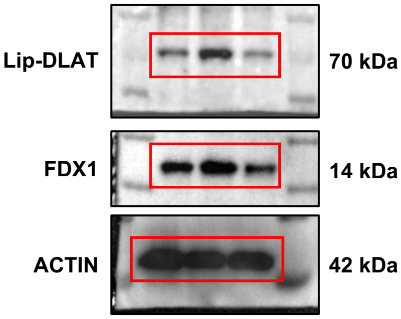

Fig. 3H

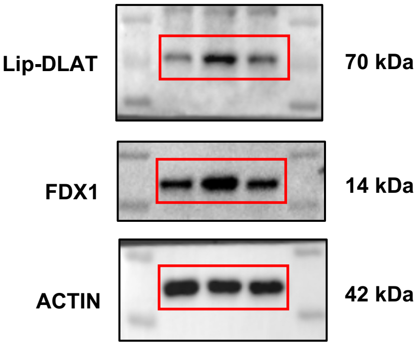

Fig. 3I

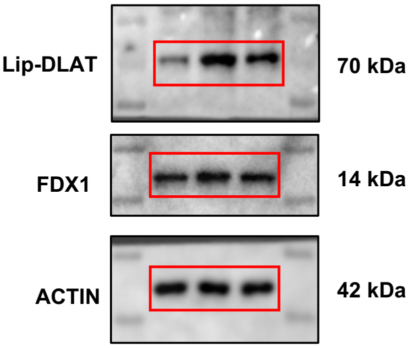

Fig. 3J

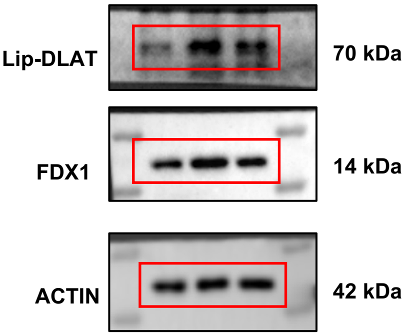

Fig. 3O

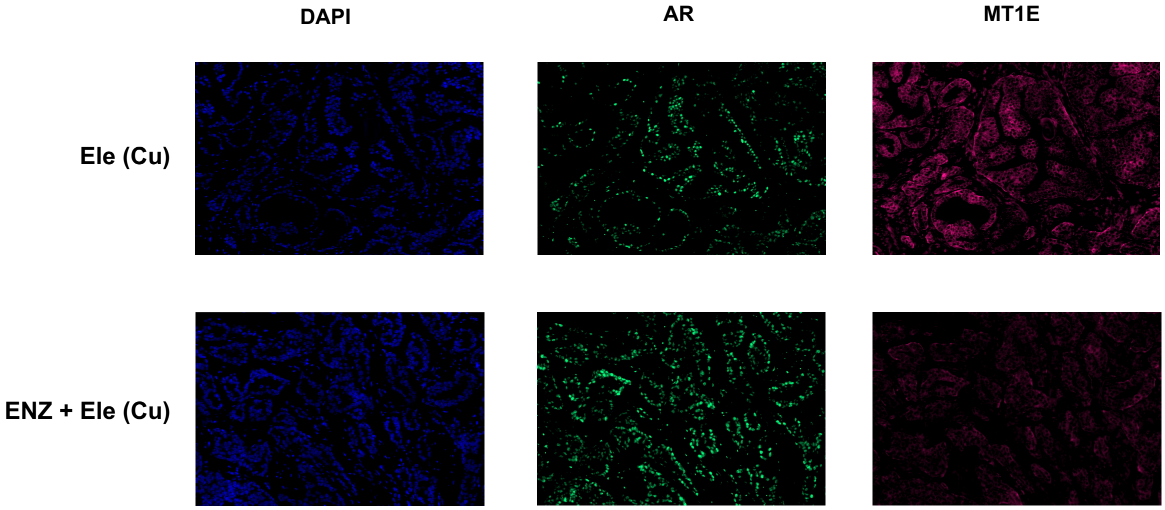
DAPI
AR
MT1E
Ele (Cu)
ENZ + Ele (Cu)

Fig. 4N

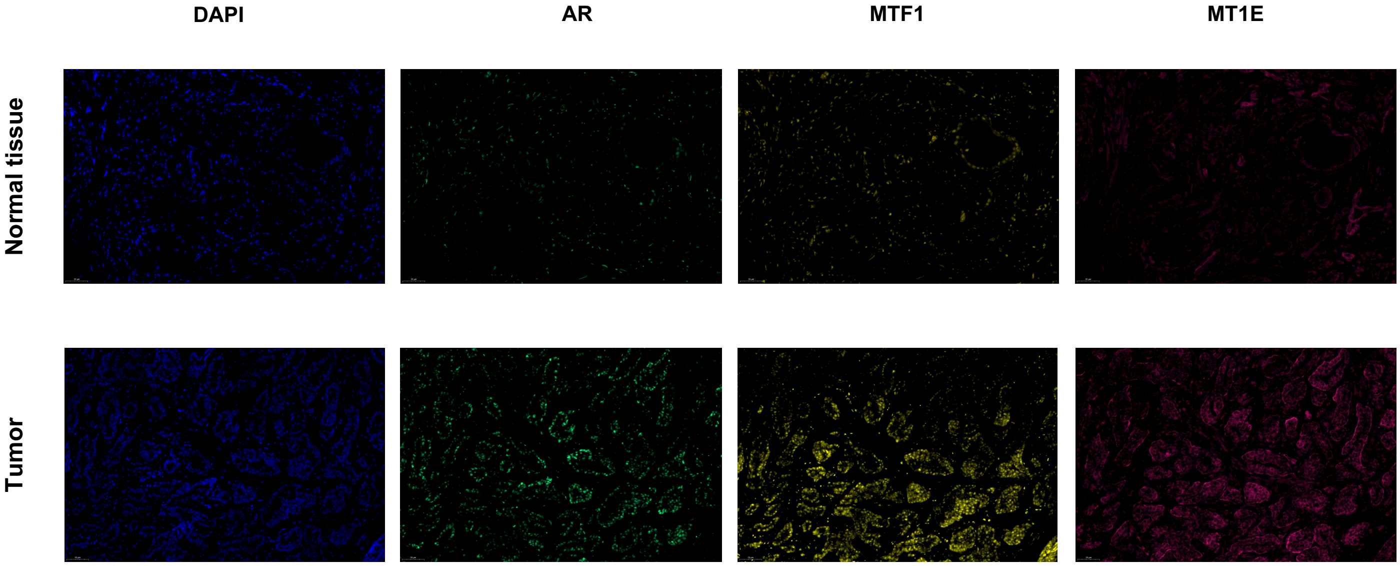
DAPI
AR
MTF1
MT1E
Normal tissue
Tumor

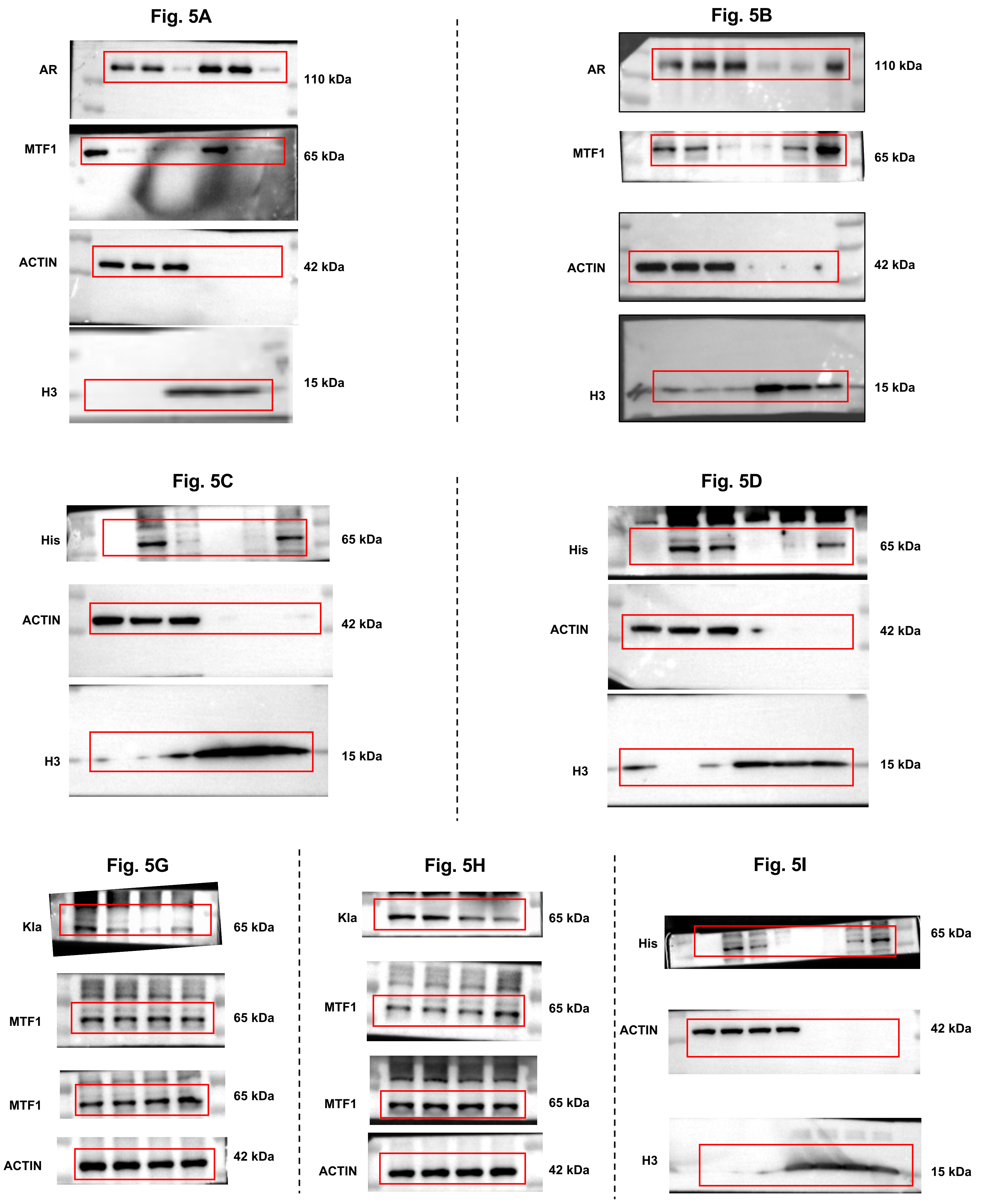

Fig. 5A
AR
110 kDa
MTF1
65 kDa
ACTIN
42 kDa
H3
15 kDa
Fig. 5B
AR
110 kDa
MTF1
65 kDa
ACTIN
42 kDa
H3
15 kDa
Fig. 5C
His
65 kDa
ACTIN
42 kDa
H3
15 kDa
Fig. 5D
His
65 kDa
ACTIN
42 kDa
H3
15 kDa
Fig. 5G
Kla
65 kDa
MTF1
65 kDa
MTF1
65 kDa
ACTIN
42 kDa
Fig. 5H
Kla
65 kDa
MTF1
65 kDa
MTF1
65 kDa
ACTIN
42 kDa
Fig. 5I
His
65 kDa
ACTIN
42 kDa
H3
15 kDa

Fig. 6A

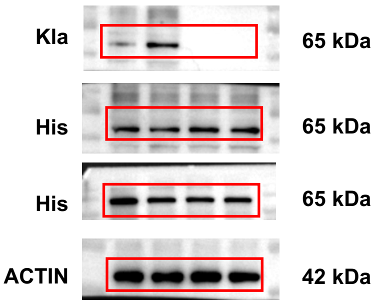

Fig. 6B

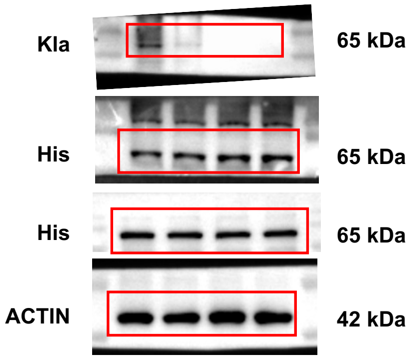

Fig. 6C

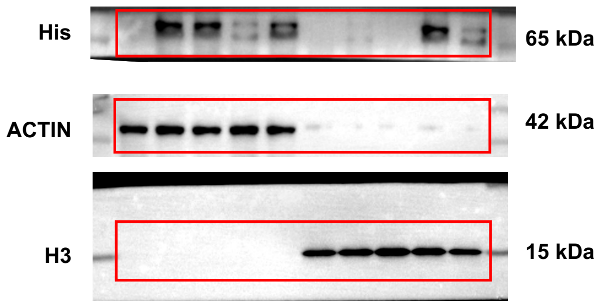

Fig. 6H

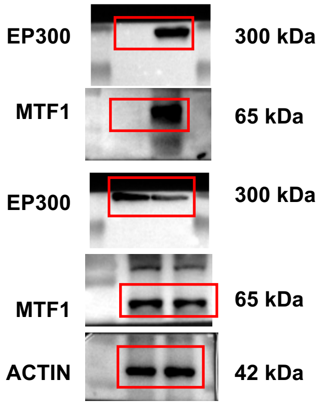

Fig. 6I

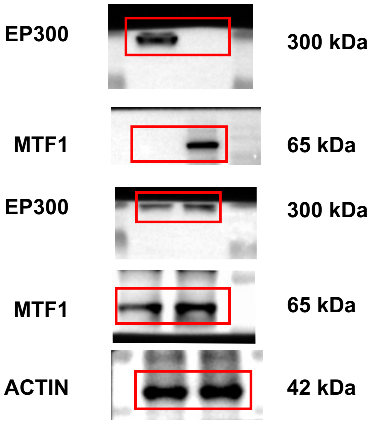

Fig. 6J

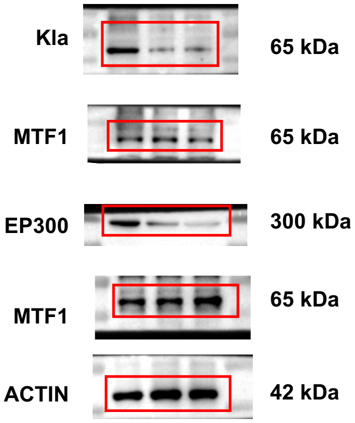

Fig. 6K

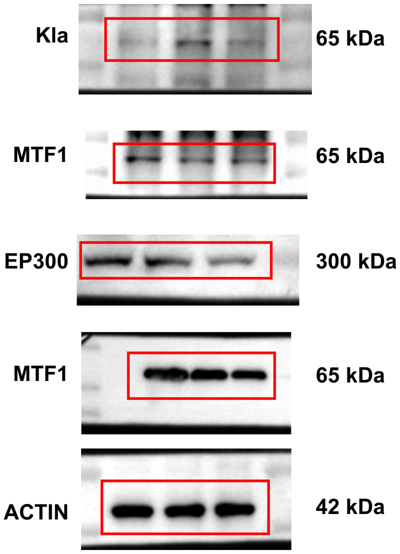

Fig. 7H

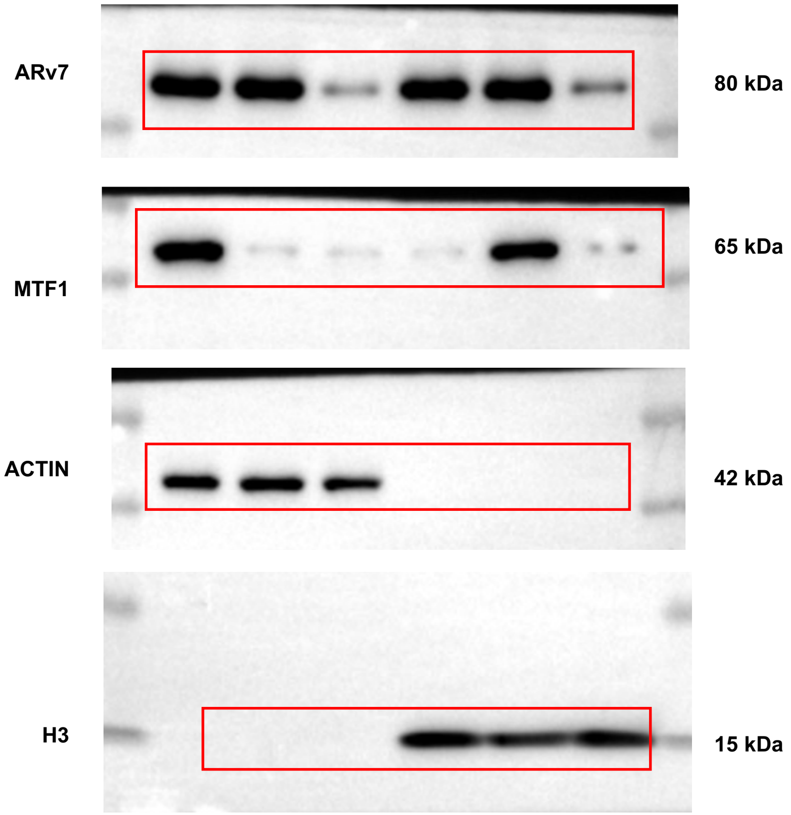

Fig. 7I

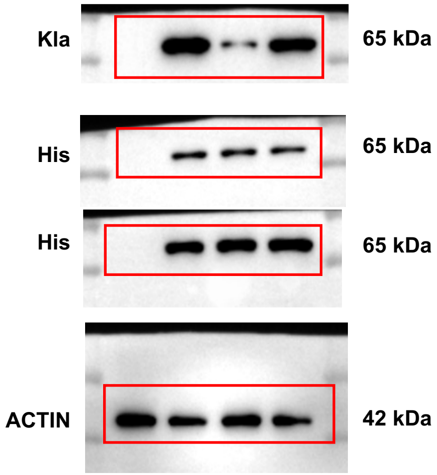

Fig. 7J

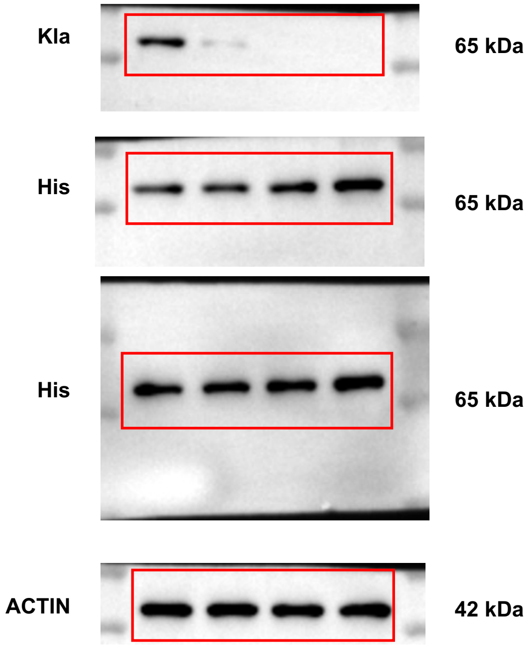

Fig. 7K

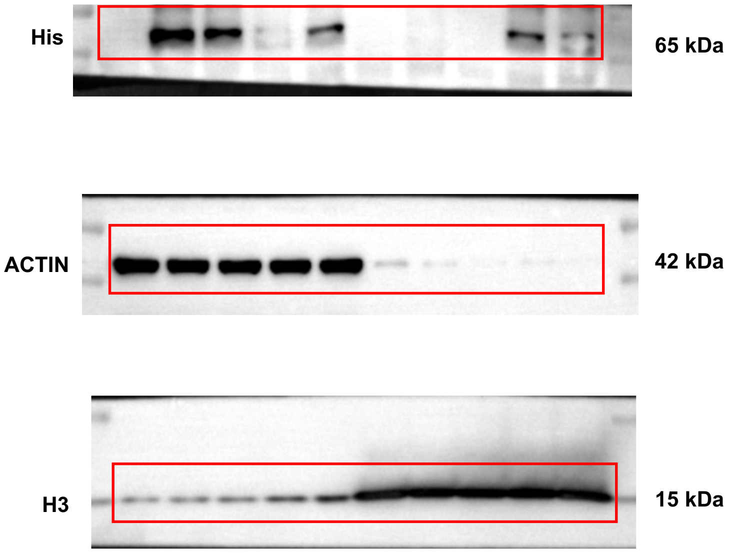

Fig. 8G

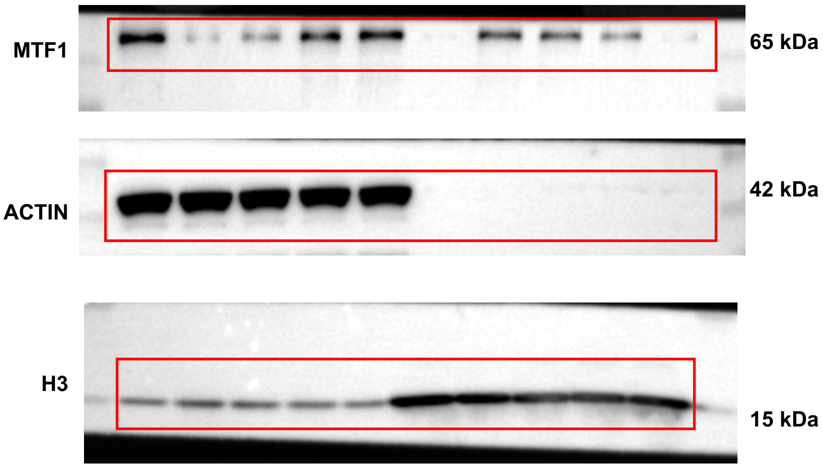

Fig. 8H

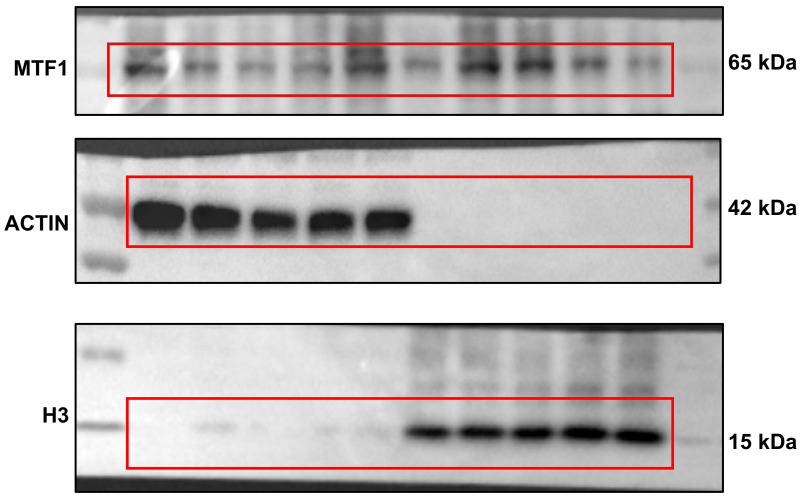

Fig. 8M

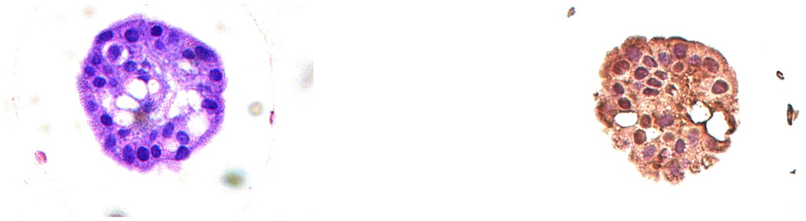

Fig. 8N

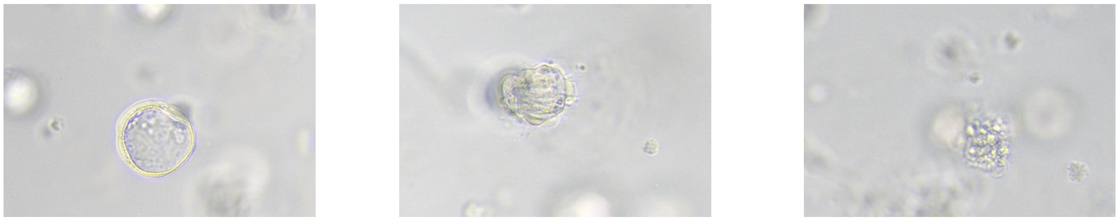

Fig. S2A

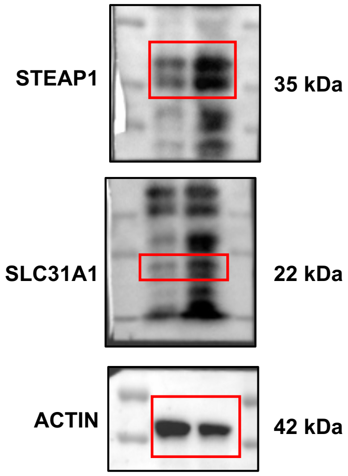

Fig. S2B

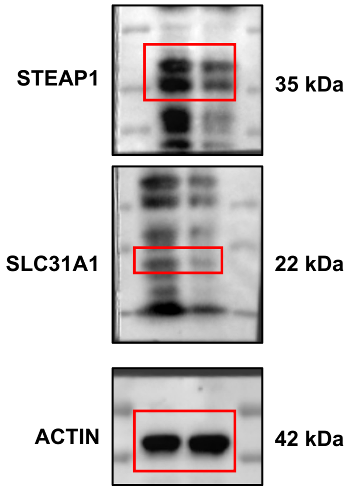

Fig. S3E

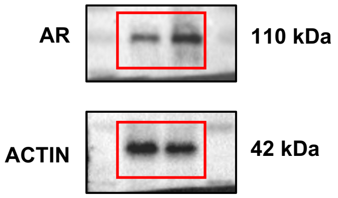

Fig. S3J

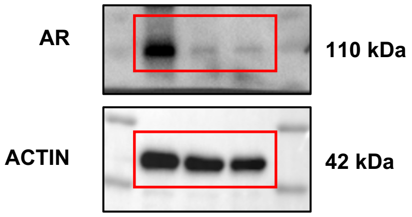

Fig.S4A

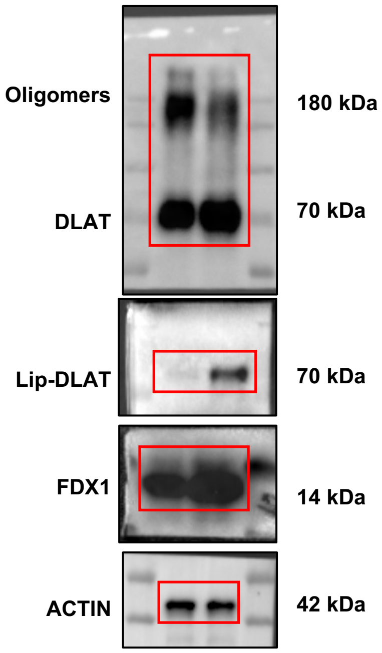

Fig. S4B

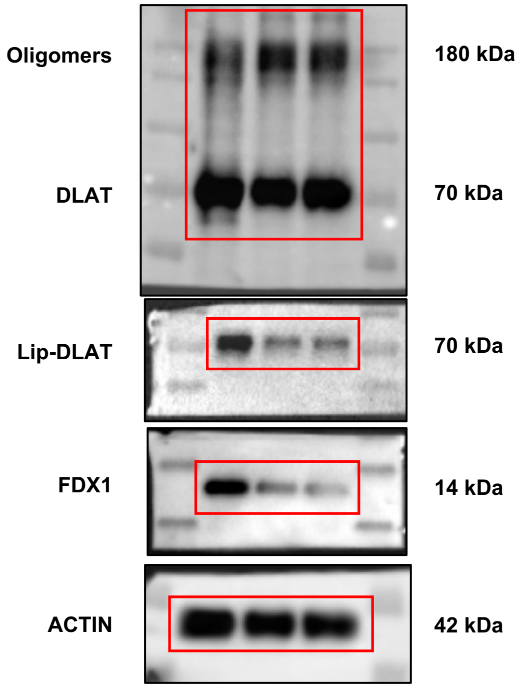

Fig. S4E

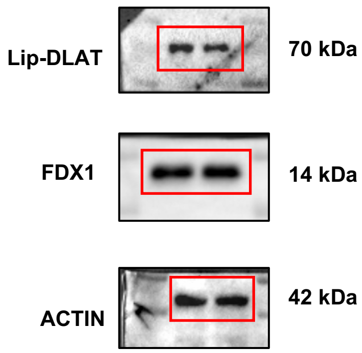

Fig. S4F

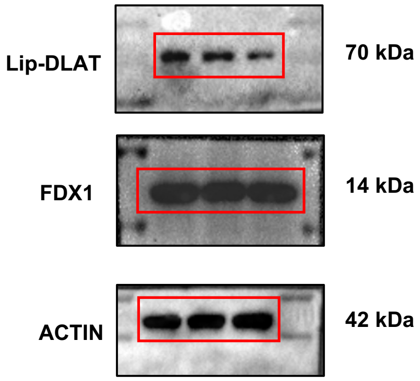

Fig. S4I

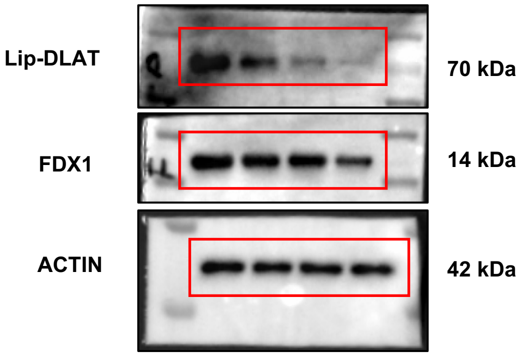

Fig. S4J

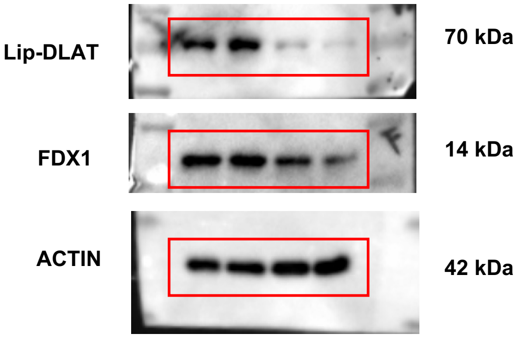

Fig. S6F

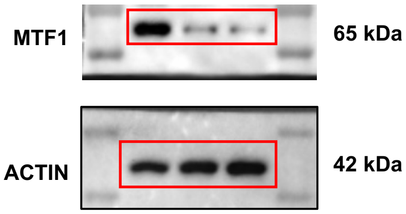

Fig. S6G

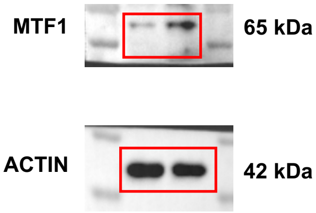

Fig.S7A

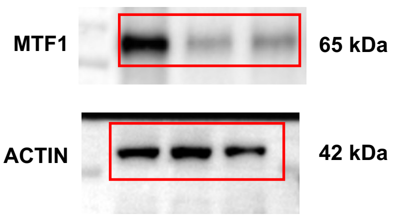

Fig. S7B

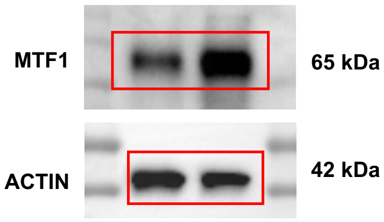

Fig. S7J

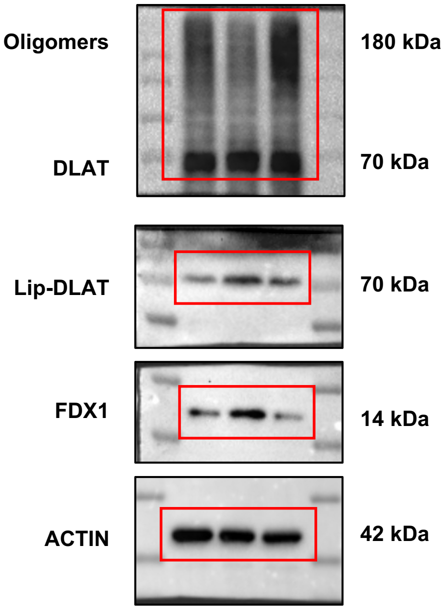

Fig. S7K

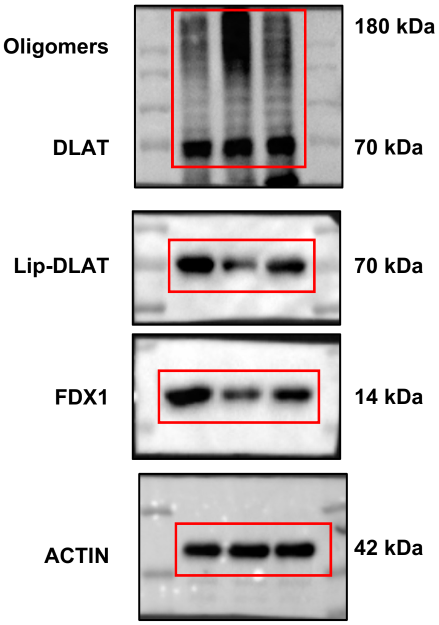

Fig. S8C

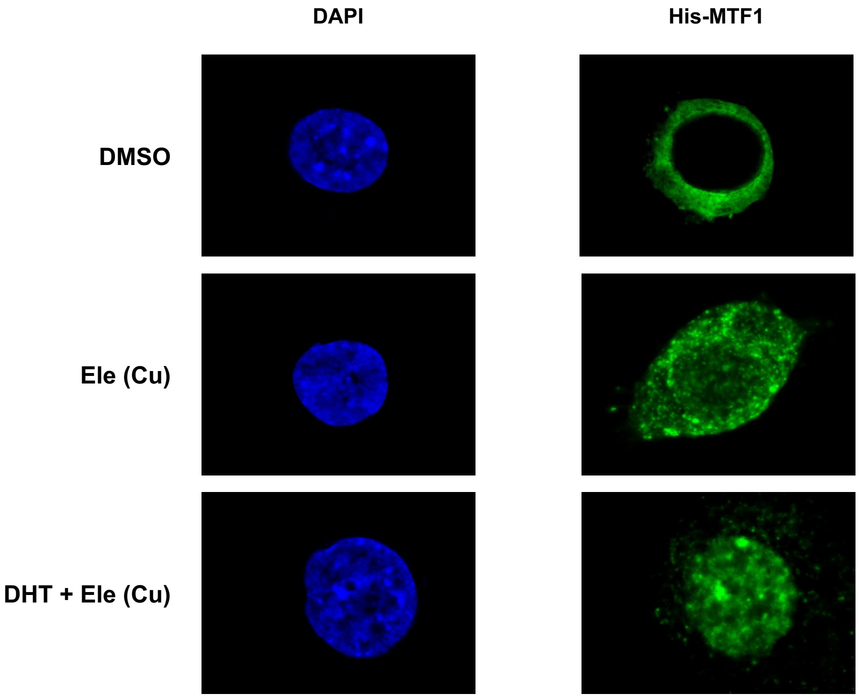

Fig. S10G

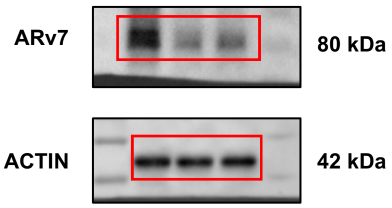

Fig. S10J

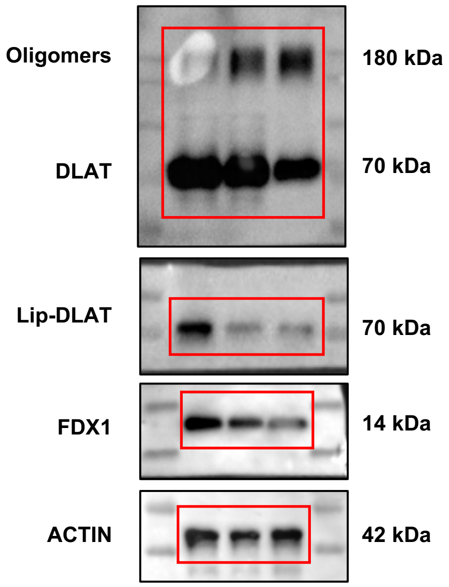

Fig. S10N

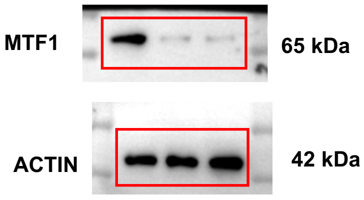

Fig. S11F

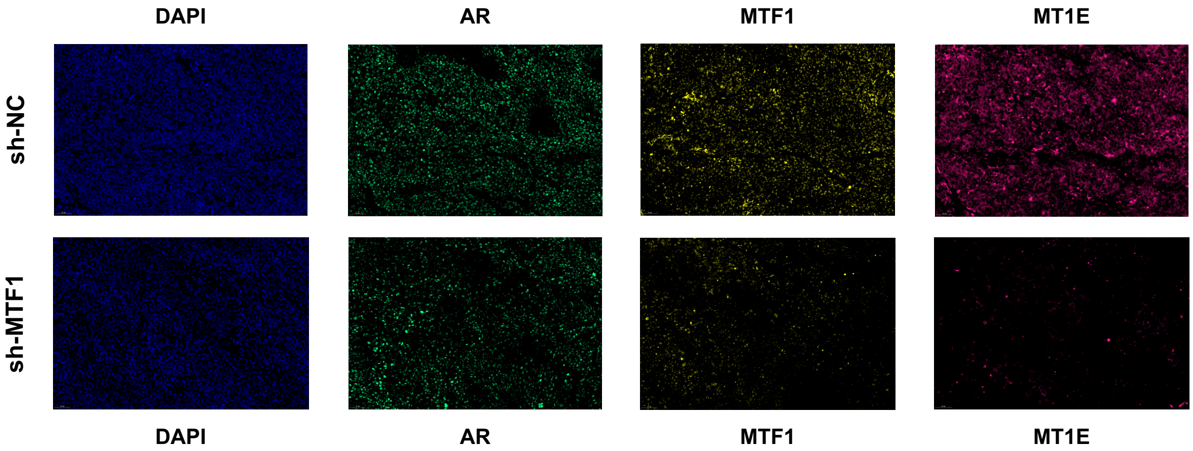

Supplement: Supplementary file 2 [file ADVS-13-e77110-s002.zip › advs77110-sup-0002-Data/rawdata.pdf]
